# Advances in the mechanism of small nucleolar RNA and its role in DNA damage response

**DOI:** 10.1186/s40779-024-00553-4

**Published:** 2024-08-08

**Authors:** Li-Ping Shen, Wen-Cheng Zhang, Jia-Rong Deng, Zhen-Hua Qi, Zhong-Wu Lin, Zhi-Dong Wang

**Affiliations:** 1 Department of Radiobiology, Beijing Key Laboratory for Radiobiology, Beijing Institute of Radiation Medicine, Beijing, 100850 China; 2 Graduate Collaborative Training Base of Academy of Military Sciences, Hengyang Medical School, University of South China, Hengyang, 421001 Hunan China

**Keywords:** Small nucleolar RNAs (snoRNAs), DNA damage response (DDR), Oxidative stress, Cell cycle checkpoints, DNA damage repair, Cell death

## Abstract

Small nucleolar RNAs (snoRNAs) were previously regarded as a class of functionally conserved housekeeping genes, primarily involved in the regulation of ribosome biogenesis by ribosomal RNA (rRNA) modification. However, some of them are involved in several biological processes via complex molecular mechanisms. DNA damage response (DDR) is a conserved mechanism for maintaining genomic stability to prevent the occurrence of various human diseases. It has recently been revealed that snoRNAs are involved in DDR at multiple levels, indicating their relevant theoretical and clinical significance in this field. The present review systematically addresses four main points, including the biosynthesis and classification of snoRNAs, the mechanisms through which snoRNAs regulate target molecules, snoRNAs in the process of DDR, and the significance of snoRNA in disease diagnosis and treatment. It focuses on the potential functions of snoRNAs in DDR to help in the discovery of the roles of snoRNAs in maintaining genome stability and pathological processes.

## Background

The precise expression of genomic DNA is crucial for all biological processes in vivo. However, cellular genomic DNA constantly faces attack, with an average of 10^4^ – 10^5^ DNA damage events occurring spontaneously in a single cell every day, posing a serious threat to genomic stability [1]. Various endogenous factors [such as DNA replication stress, reactive oxygen species (ROS) generated by cellular respiration and lipid peroxidation, and other factors produced by physiological metabolism] and multiple exogenous factors [including ultraviolet (UV) radiation, ionizing radiation, genotoxic chemicals, and viruses] can cause damage to DNA bases or molecular structure of DNA. Types of DNA damage include base insertions/deletions, mismatches, interstrand crosslinks, single-strand breaks (SSBs), and double-strand breaks (DSBs). To counteract such damage, eukaryotic organisms have developed a highly conserved system known as the DNA damage response (DDR), which undergoes precise regulation through a series of biochemical processes within the cell. In addition to regulating biological processes involved in repairing damaged DNA, the DDR system modulates other processes such as cell cycle progression, chromatin remodeling, gene transcription activity, and cell death. This system plays a vital role in maintaining genomic stability while enhancing cell tolerance to DNA damage and triggering death mechanisms in cells with damaged genomes [2, 3]. Furthermore, the dysfunction of the DDR system leading to genome instability is closely associated with various diseases, including cancer, neurodegenerative diseases, immune deficiencies, inflammation, aging, cardiovascular diseases, and metabolic disorders [3–8].

Small nucleolar RNAs (snoRNAs) are a class of small non-coding RNA (ncRNA) molecules primarily located in the nucleolus of cells. They typically consist of 60 – 300 nucleotides (nt) and were first discovered in mammals during the late 1960s and early 1970s [9]. The rapid development of high-throughput sequencing technologies has facilitated the identification of numerous snoRNAs in the human genome [10, 11]. Previously considered functionally conserved housekeeping genes primarily involved in ribosome biogenesis, recent research has revealed that snoRNAs possess diverse biological functions and regulate various processes, including individual lifespan, neurodevelopment, cardio-cerebrovascular diseases, stem cell differentiation, metabolism, hematopoiesis, immunity, and stress reactions [12–20]. While several other ncRNAs, including microRNAs (miRNAs), long ncRNAs (lncRNAs), and circular RNAs (circRNAs) have been extensively validated for their roles in regulating DNA damage repair and genome stability [21], only a limited number of snoRNAs involved in this process have been identified thus far. Further investigations have provided evidence suggesting that multiple snoRNAs participate in biological processes closely related to DDR, such as oxidative stress, cell cycle progression, inflammation, and immunity, as well as cell death; however, the underlying mechanisms by which these snoRNAs regulate these processes remain unclear. Despite previous studies conducted on this topic, the full extent to which snoRNA is involved in DDR remains unknown [18, 22].

With recent advancements in our understanding of the biological functions of snoRNAs, this current review aims to provide a comprehensive summary of their biological characteristics and elucidate the mechanisms by which they regulate target molecules. Furthermore, we highlight the potential roles of snoRNAs in DDR and shed light on their underlying mechanisms involved in DDR regulation, thus clarifying their significance in the maintenance of genomic stability during DDR.

## Biosynthesis, molecular structure and classification of snoRNAs

**Fig. 1:**
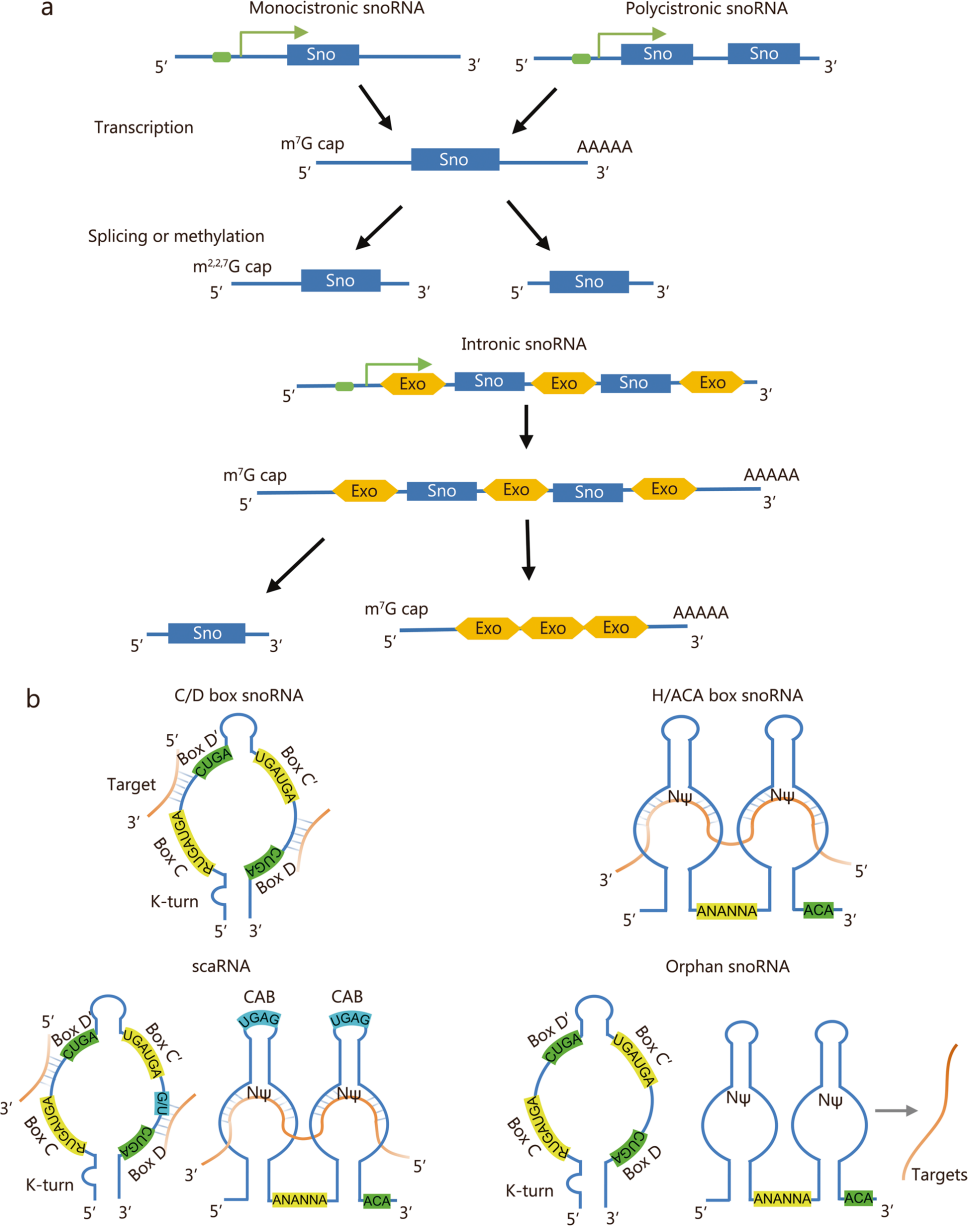
Synthetic mechanism and classification of small nucleolar RNAs (snoRNAs). **a** Biosynthesis of snoRNAs. **b** Schematic diagram of snoRNA structure and classification. Sno snoRNA, Exo exons, NΨ nucleotides modified by psuedouridylation, CAB small Cajal body localization element

SnoRNAs are highly conserved across eukaryotes, including mammals, African-clawed frogs, plants, and yeast. However, their number, arrangement in the genome, and transcription mechanisms vary among different species. In unicellular eukaryotes like brewer’s yeast, most snoRNA genes exist independently from the genome and are transcribed as monocistrons. In contrast, fruit flies and nematodes typically have snoRNAs arranged in a multigenic manner and transcribed as polycistrons. In humans, snoRNAs are primarily located in the intronic regions of coding or non-coding host genes and co-transcribed with them [23]. While the majority of snoRNAs are transcribed by RNA polymerase II, a small number are transcribed by RNA polymerase III. Notably, certain snoRNA molecules such as *U3* in yeast and animals are transcribed by RNA polymerase II while being transcribed by RNA polymerase III in plants [24]. The subsequent processing of primary transcripts to mature snoRNA molecules involves a series of biological processes, such as removing the m^7^G cap at the 5’ end or (alternatively) converting it into a 2,2,7-trimethylguanosine (m^2,2,7^G) cap [25–29] (Fig. 1a).

In general, snoRNAs are divided into 2 classes based on their conserved sequence elements: C/D box and H/ACA box snoRNAs. C/D box snoRNAs consist of a 5’ terminal C box (5’-RUGAUGA-3’, where R represents A or G) and a 3’ terminal D box (5’-CUGA-3’). Most C/D box snoRNA molecules also contain additional sequence elements that are similar to the C and D boxes (usually with slight variations of 1 – 2 nt), known as the C’ and D’ boxes (Fig. 1b). Additionally, most boxes C/D have reverse-repeat sequences consisting of 4 – 5 nt as their ends. Notably, the presence of the C box at the 5’ end and the D box at the 3’ end, along with their corresponding terminal pairing sequences, form a conserved “stem-bulge-stem” secondary structure referred to as the “kink-turn” or “K-turn”. This structure serves as the functional core of a small nucleolar ribonucleoprotein (snoRNP) complex containing a C/D box. The formation of these complexes involves multiple ribonucleoproteins binding to them, including 2’-O-methyltransferase fibrillarin (homologous to the yeast protein Nop1p), Nop56, Nop58 (homologous to the yeast protein Nop5p), and p15.5KD (homologous to the yeast protein Snu13p) [25, 30–32]. By contrast, H/ACA box snoRNAs adopt a conserved “hairpin-hinge-hairpin-tail” structure. The box H (5’-ANANNA-3’, where N represents any nt) is located in the single-stranded hinge region while the box ACA (sometimes AUA) is generally positioned 3 nt upstream from its 3’ end (Fig. 1b). Some organisms such as archaea generate H/ACA box snoRNAs with either single hairpin or double hairpin structures [33]. Typically, H/ACA box snoRNP complexes form by associating pseudouridine synthase dyskerin (homologous to the yeast protein Cbf5p), Gar1p, Nhp2p, and Nop10p.

In addition to the C/D box and H/ACA box snoRNAs, the cell contains several specialized snoRNAs. Small Cajal body-specific RNAs (scaRNAs) are a subset of the snoRNA family. As a general rule, scaRNAs exhibit both structural and functional characteristics of C/D box or H/ACA box snoRNAs (in some cases, they contain both C/D box and H/ACA box sequences). In addition, scaRNAs possess a small Cajal body localization element (CAB box) (a GU repeat sequence in C/D box scaRNA and 5’-UGAG-3’ sequence in H/ACA box scaRNA). The presence of the CAB box enables specific translocation of scaRNAs to the Cajal bodies, where they target and modify small nuclear RNAs (snRNAs), participating in the assembly of spliceosomes [34, 35]. However, there are several orphan snoRNAs have been identified without definite complementary sequences or target molecules discovered yet (Fig. 1b). A small number of these orphan snoRNAs play essential roles in tissue development, cardiovascular diseases, tumors, and other pathological processes. Nevertheless, the biological functions of most orphan snoRNAs remain unknown [14, 36–38]. According to existing literature, a schematic description illustrating the synthesis mechanisms and the classification of snoRNAs was prepared (Fig. 1) [23, 25, 35, 39].

## Mechanisms through which snoRNAs regulate target molecules

**Fig. 2:**
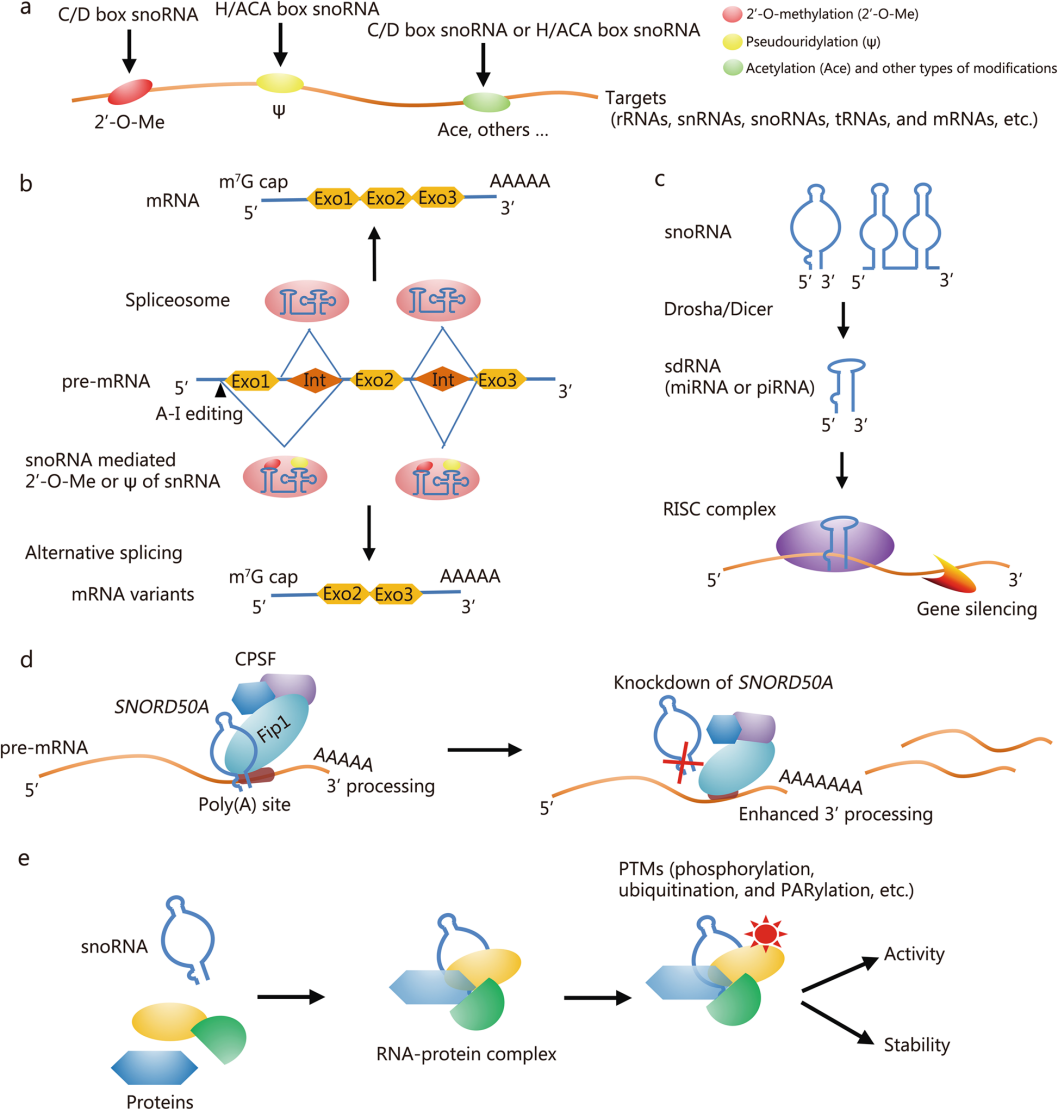
Mechanisms through which small nucleolar RNAs (snoRNAs) interact with targets.** a** snoRNAs facilitate the post-transcriptional modifications of certain RNA molecules. **b** snoRNA-mediated alternative splicing by adjusting the activity of spliceosome or RNA editing. **c** snoRNA-derived small RNA fragments (sdRNAs) mediate gene silencing, functioning as miRNAs or piRNAs. **d** Involvement of snoRNAs during the pre-mRNA processing, with *SNORD50A* as an example. *SNORD50A* directly interacts with Fip1, a core component of cleavage and polyadenylation specificity factor (CPSF) at the poly(A) site to maintain moderate mRNA 3’ processing efficiency, and *SNORD50A* knockdown leads to process disruption. **e** snoRNAs regulate post-translational modifications (PTMs) through RNA-protein interactions, leading to changes in the activity and stability of certain proteins. Eon exon, Int intron, rRNA ribosomal RNA, snRNA small nuclear RNA, tRNA transfer RNA, mRNA messenger RNA, miRNA microRNA, piRNA Piwi-interacting RNA, RISC RNA-induced silencing complex, A-I adenine to inosine

Previously, it was believed that snoRNAs exclusively regulate RNA 2’-O-methylation (2’-O-Me) and pseudouridylation by binding to target RNA molecules through complementary base pairing. However, in-depth study on the functions and mechanisms of snoRNAs has revealed diverse modes of action [40]. Indeed, recent research has unveiled several novel aspects [15, 41–43]. Firstly, the target molecules of snoRNAs are not limited to ribosomal RNAs (rRNAs) and snRNAs but also encompass other types such as messenger RNAs (mRNAs) and transfer RNAs (tRNAs). Moreover, the regulation role of snoRNA extends beyond 2’-O-Me and pseudouridylation to include acetylation modification in post-transcriptional RNA regulation. Secondly, gene expression is modulated by snoRNAs through derived small RNA fragments known as sno-derived RNAs (sdRNAs). Thirdly, snoRNAs play a role in regulating RNA editing and alternative splicing processes. Finally, interaction with proteins allows for the regulation of their stability and activity by snoRNAs (Fig. 2).

### Regulation of post-transcriptional modifications

In the classic functional mechanism model, snoRNAs regulate the post-transcriptional modification of RNA molecules through base-complementarity pairing. Generally, their downstream targets are pre-rRNA, rRNAs, or snRNAs. However, whole transcriptome sequencing analyses have revealed that other types of RNA molecules, such as snoRNAs, mRNAs, and tRNAs, typically contain target sites modified by snoRNAs (Fig. 2a). The appropriate modification of key bases has a significant impact on the folding, processing, and functional site recognition of these targeted RNAs [41]. Research has focused on the mechanisms by which C/D box snoRNAs mediate 2’-O-Me modifications and H/ACA box snoRNAs promote pseudouridylation in rRNA [23]. C/D box snoRNAs recognize target molecules via the anti-sense element adjacent to boxes D and D’, guiding the 2’-O-Me together with the aid of certain proteins, such as fibrillarin (Nop1p), Nop56, Nop58 (Nop5p), and p15.5KD (Snu13p). Conversely, H/ACA box snoRNAs bind to target sequences through hairpin structures, promoting isomerization of uridine residues into pseudouridine with help from core factors such as dyskerin (Cbf5p), Gar1p, Nhp2p, and Nop10p [25, 40, 43]. Hairpin structures within H/ACA box snoRNAs contain pseudouridylation pockets facilitating this reaction. Each pocket contains one or two guide sequences that pair via base-complementarity with the sequence at the target site (usually 4 – 8 nt). The pseudouridylation site generally lies between boxes H and ACA approximately 14 – 16 nt upstream, and this distance is the key factor in selecting the correct modification site [44, 45]. To our knowledge, the process of the snoRNA-mediated 2’-O-Me and pseudouridylation in pre-rRNA/rRNA represents one of the most crucial regulatory mechanisms of ribosome biosynthesis.

A disruption in snoRNA-mediated RNA 2’-O-Me and pseudouridylation significantly impacts the process of protein translation. McMahon et al. [46] discovered that *SNORA24* in hepatoma cells targets the pseudouridylation of 18S rRNA at the U609 and U863 sites, facilitating accurate recognition of mRNA codons by aminoacyl-tRNA. Thus, the inhibition of *SNORA24* expression and activity led to an increase in translation error rate for certain specific mRNA molecules. The direct modification of mRNAs mediated by snoRNA is also an important pathway for regulating translation. A report in *Nature* in 2011 by Karijolich et al. [47] provided evidence that the snoRNA *snR81-1C* regulates mRNA pseudouridylation. The authors found that incorrect mRNA modification leads to codon decoding errors, and false pseudouridylation of stop codons (termination codons) unexpectedly endowed them with encoding abilities. Subsequently, Elliott et al. [48] reported that the C/D box snoRNAs *SNORD32A* and *SNORD51* in human cells, which are encoded by ribosomal protein L13a (rpL13a), target *peroxidasin* mRNA with the assistance of the fibrillarin protein. This process results in the 2’-O-Me of the adenosine residue A3150 in the coding region, increasing the stability of the mRNA. As the base modification creates a spatial barrier that interferes with the interaction between rRNA and the mRNA-tRNA minihelix phosphate ribose backbone, it significantly inhibits the translation efficiency of *peroxidasin* mRNA [40, 48, 49]. Likewise, van Ingen et al. [50] reported that *SNORD113* targets various mRNA molecules to regulate their stability by adjusting 2’-O-Me. Therefore, the snoRNA-mRNA interaction that occurs within cells may not be coincidental events. In addition to rRNAs and mRNAs, snoRNAs participate in the process of protein translation by regulating tRNA methylation. According to reports by Vitali et al. [51], *SNORD97* and *SCARNA97* jointly regulate 2’-O-Me at the C34 position of tRNA^Met^ (CAT), protecting it from degradation caused by nucleic acid endonucleases under stressful conditions. Hence, it has been argued that the snoRNA-mediated modulation of the translation process is not only a novel mechanism for generating protein diversity but also a critical factor contributing to mutations, erroneous protein accumulation, and increased susceptibility to diseases [46, 47].

Additional research on snoRNAs regulating the modification of other types of RNAs is sparsely documented. In 2017, Sharma et al. [42] reported a new pathway through which snoRNAs regulate post-transcriptional modification, specifically snoRNA-mediated acetylation. The authors found that *snR4* and *snR45* in yeast cells facilitate the acetylation of 18S rRNA under the catalytic influence of acetyltransferase Kre33 (homologous to human N-acetyltransferase 10 protein), thereby regulating the processing of pre-rRNA. Subsequently, Bortolin-Cavaillé et al. [52] and Thalalla Gamage et al. [53] confirmed that *SNORD13* in human cells, a homolog of *snR45*, also mediates 18S rRNA acetylation. To date, numerous RNA modifications have been identified with some only recently gaining recognition for their significance. For example, there has been a notable surge in research interest regarding N^6^-methyladenine methylation [54]. Thus, it is reasonable to deduce that snoRNAs are implicated in other types of RNA modifications (Fig. 2a). These studies suggest that the widespread impact of snoRNAs on post-transcriptional modifications may surpass current understanding.

### Regulation of RNA alternative splicing

RNA splicing, which includes both constitutive and alternative splicing, is a biological process wherein the introns are removed from the primary transcription products (e.g., pre-mRNA) under the catalysis of spliceosomes, and the exons are joined together to yield mature RNA molecules (e.g., mRNA). This process has significant implications for maintaining the diversity, tissue-specificity, and spatiotemporal specificity of gene expression in eukaryotes. Approximately 90 – 95% of multi-exon gene transcripts in the human body undergo alternative splicing. Importantly, defects in this process can lead to diseases such as cancer and certain genetic developmental disorders [55–57]. Several conserved nt sequences facilitate the recruitment of specific spliceosomes and related regulatory factors such as heterogeneous nuclear ribonucleoproteins and serine/arginine-rich proteins during the pre-mRNA splicing process. These factors recognize the splice sites and catalyze two transesterification reactions to remove the introns and connect the exons. The conserved sequences necessary for this process include the 5’ splice site, 3’ splice site, branch point (situated 18 – 40 nt upstream of the 3’ splice site), polypyrimidine tract, and multiple cis-acting elements (enhancers and silencers). The spliceosome itself consists of 5 nuclear ribonucleoprotein (snRNPs) U1, U2, U4, U5, and U6, each containing a specific snRNA and bound Smith antigen (Sm) proteins that share a conserved bipartite Sm motif or ‘‘Sm fold’’ [58, 59]. The assembly of the spliceosome begins with U1 recognizing the 5’ splice site and U2 recognizing the branch point. The subsequent recruitment of the U4/U5/U6 polymer to U1/U2 triggers a conformational change in proteins, and exchange of protein molecules, and ultimately leads to the formation of a catalytically active spliceosome. Mutations in core components of the spliceosomes such as U2 small nuclear RNA auxiliary factor 1 (U2AF1), serine/arginine-rich splicing factor 2 (SRSF2), splicing factor 3b subunit 1 (SF3B1), and U1 result in aberrant RNA splicing. These mutations have been extensively identified in cancers, highlighting their significance as therapeutic targets [57, 60]. Apart from these factors mentioned above, RNA modifications, RNA editing, and RNA secondary structure also play crucial roles in alternative splicing. Particularly, ADAR-mediated adenine to inosine (A-I) editing (which enables the recognition of “I” as “G” by some cellular machinery) facilitates the formation of novel splice sites in the RNA sequence and consequently generates new mRNA molecule subtypes with distinct functions [61, 62].

The importance of snoRNAs in the regulation of RNA alternative splicing is now being widely recognized (Fig. 2b). snoRNAs precisely orchestrate a series of RNA-RNA and RNA-protein interactions to regulate spliceosome assembly, splice site recognition, and the RNA editing process. Methylation or pseudouridylation modifications mediated by snoRNA at specific snRNA sites is an important step that affects snRNP synthesis, spliceosome assembly, and spliceosome localization. It is reported that most of the small RNA molecules that regulate snRNAs U1, U2, U4, U5, and U6 (and related molecules) are scaRNAs [34, 35]. Furthermore, alterations in the expression of several scaRNAs known to regulate snRNA modifications (e.g., *scaRNA1* and *scaRNA15*) occur during cardiovascular development and carcinogenesis, leading to abnormal mRNA splicing [63, 64]. However, the snoRNA-mediated regulation of RNA splicing is not always dependent on the classical snRNA 2’-O-Me and pseudouridylation modifications; some snoRNAs bind to cis-acting elements in mRNAs while competing with spliceosome proteins for binding sites. Falaleeva et al. [65] analyzed snoRNAs in different nuclear components and identified approximately 30 types of snoRNAs enriched by spliceosome-related proteins. They subsequently explored the role of one particular snoRNA called *SNORD27* in the regulation of early 2 factor (*E2F*) transcription factor 7 (*E2F7*) pre-mRNA splicing. The authors found that *SNORD27* knockdown promotes the expression of a silenced exon in the target molecule. Based on their results, they postulated that *SNORD27* competitively binds to the splice site in the target mRNA through an independent 2’-O-Me process via RNA-RNA pairing instead of directly interacting with snRNP U1.

Another classic example of snoRNAs regulating alternative splicing involves *SNORD115* (also known as *HBII-52*). The loss of *SNORD115* expression is a pivotal genetic factor leading to the development of Prader-Willi syndrome (PWS) [13]. The aberrant expression of the transmembrane protein 5-hydroxytryptamine 2C receptor (5-HT_2C_R) is strongly associated with the pathological progression of PWS. The V exon of *5-HT*_*2C*_*R* pre-mRNA undergoes alternative splicing to produce two subtypes: Va and Vb. The intracellular loop encoded by Vb encompasses a crucial domain for G protein signaling, and skipping of Vb significantly reduces the responsiveness of 5-HT_2C_R to serotonin. Moreover, A-I editing in Vb promotes the expression of Vb-type 5-HT_2C_R; however, alterations in the amino acid sequence within the intracellular loop reduce its activity. Kishore et al. [66] found that *SNORD115* binds to the silent element in *5-HT*_*2C*_*R* pre-mRNA Vb through base pairing, thereby inhibiting Vb exon skipping caused by alternative splicing. Additionally, *SNORD115* exerts an inhibitory effect on the A-I editing of *5-HT*_*2C*_*R* pre-mRNA, thus preventing excessive RNA editing and subsequent synthesis of low-activity 5-HT_2C_R proteins. Doe et al. [67] and Glatt-Deeley et al. [68] reported a significant increase in A-I editing levels in *5-HT*_*2C*_*R* pre-mRNA within brain tissues from patients with PWS and a PWS mouse model (PWS-IC 1/2) deficient in *mbii-52* gene expression. Moreover, Bratkovič et al. [37] reported the editing frequency at the A-I editing sites (A, B, E, C, and D) in *5-HT*_*2C*_*R* pre-mRNA and observed a significantly higher frequency of editing at the unbound A and B sites compared to the bound E, C, and D sites regulated by *SNORD115*. These findings indicate substantial discrimination in the selection of A-I editing sites and suggest that *SNORD115* promotes Vb-type *5-HT*_*2C*_*R* mRNA expression independently of A-I editing. It should be noted that despite the widespread acceptance of the notion that “*SNORD115* affects 5-HT_2C_R expression via A-I editing involved in the pathogenesis of PWS”, some evidence contradicts this perspective [68].

### Mediating gene silencing

In general, regulatory small RNA molecules such as miRNAs, small interfering RNAs, and Piwi-interacting RNAs (piRNAs) are produced in cells through the cleavage and processing of double-stranded RNA by specific nucleases, including Drosha, Dicer, and related auxiliary factors. Subsequently, these regulatory small RNA molecules bind to the RNA-induced silencing complex that is centered on argonaute (Ago) proteins. These complexes selectively recognize and bind to target RNAs, resulting in either degradation or inhibition of mRNA translation and thereby mediating the silencing of gene expression (Fig. 2c). Recent studies have revealed that snoRNAs also serve as a significant source for these regulatory small RNA molecules [43, 69–71].

In 2008, Kawaji et al. [69] discovered that various types of ncRNAs, including snoRNAs, are processed in human cells to generate many small RNA fragments ranging from 20 to 40 nt in length. Subsequent studies further demonstrated that over 60% of snoRNAs in humans and mice (and even higher proportions in other species such as chickens, Drosophila, Arabidopsis, and fission yeast) are cleaved and processed into sdRNAs. The sdRNAs originating from the H/ACA box snoRNAs primarily derive from their 3’ ends and typically range from 20 to 24 nt in length, resembling classical miRNA molecules. On the other hand, the sdRNAs generated from C/D box snoRNAs mainly originate from their 5’ ends and are typically either 17 – 19 nt or 30 nt long; the shorter sdRNAs are classified as miRNAs while the longer ones are categorized as piRNAs [70] (Fig. 2c).

Although some sdRNAs can be stably expressed in cells, the current understanding of their function is extremely limited. In the case of the few known sdRNAs, their processing shares similarities with classical miRNAs and piRNAs in terms of a signaling pathway (although it does not completely depend on the expression of Drosha or Dicer), as well as biological functions [70, 72, 73]. Ender et al. [72] found that a small RNA derived from the 3’ end of snoRNA *ACA45* in human cells could bind Ago. Furthermore, this sdRNA is generated by the RNA endonuclease Dicer (not affected by Drosha/DGCR8) and targets the 3’ UTR region of *DC2L6* (*CDK11*) mRNA to inhibit its expression similar to miRNAs. Other studies found that *miR-605* (*sno-miR-605*) derived from a H/ACA box snoRNA and *sno-miR-28* derived from *SNORD28* participate in the regulation of p53 function through gene silencing [74–76]. Additionally, Zhong et al. [77] reported that *piR30840* derived from *SNORD63* binds to the intronic region of *IL4* pre-mRNA in human primary CD4^+^ T lymphocytes, subsequently recruiting the Trf4-Air2-Mtr4 complex for *IL4* mRNA degradation via the nuclear exosome pathway, thereby regulating IL4 expression and modulating Th2 lymphocyte development. However, not all sdRNAs negatively regulate their target genes. For example, *pi-sno75*, derived from *SNORD75*, regulates the methylation levels in the promoter region of tumour necrosis factor (TNF)-related apoptosis-inducing ligand (*TRAIL)*, facilitating transcription and enhancing the expression of the pro-apoptotic protein TRAIL, ultimately promoting an anti-cancer effect [78]. In brief, the discovery of sdRNAs is an important step in reconstructing the regulatory networks of ncRNAs while also increasing attention to their roles in diseases [71, 79, 80].

### snoRNA interaction with proteins

Generally, RNA-protein interactions are a prevalent mechanism by which ncRNAs exert their functions, and snoRNAs are no exception. snoRNAs function as molecular scaffolds to promote the formation of RNA-protein complexes, regulate the interactions between protein subunits to modulate protein activities, and act as molecular guides for directing proteins in recognition and anchoring target molecules [40]. This mode of action is extremely important for the snoRNA-mediated regulation of rRNA modification and ribosome biogenesis. Building upon this principle, Song et al. [81] developed snoRNAs as tool vectors for inducing specific modifications at RNA locus to re-edit disease-associated aberrant mRNAs, thereby enhancing gene expression.

As the diverse functions of snoRNAs are gradually being revealed, it is evident that their interactions with proteins have a significant impact on a wide range of biological processes. Specifically, they regulate gene expression at multiple levels by controlling mRNA terminal processing, translation, and post-translational modification (PTM). The addition of a 5’ “capping” and 3’ “poly(A)” tail during the pre-mRNA processing is crucial for mRNA maturation as it prevents degradation by nucleases. Huang et al. [82] analyzed RNAs enriched in mRNA 3’ terminal processing complexes and found that most were snoRNAs. Importantly, the authors confirmed the direct binding between *SNORD50A* and Fip1 protein (a core component of the terminal processing complex) both in vitro and in vivo. By directly binding to the Fip1 protein, *SNORD50A* inhibits the interaction between Fip1 and the poly(A) site at the 3’ ends of mRNAs, thereby preventing excessive polyadenylation which regulates the expression of various mRNAs (Fig. 2d). Oncology research has revealed that the direct interaction between snoRNAs and tumor suppressor proteins regulates the activity of cancer signaling pathways, which has a significant impact on cellular transformation and tumor progression. For example, *SNORD6* enhances the interaction between the E6/E6AP complex and p53 protein by binding to the E6 protein, subsequently promoting the ubiquitination and degradation of the p53 protein [83]. The snoRNA *SNORD50A/B* binds to K-Ras and regulates its C-terminal farnesylation modification, thereby inhibiting the activation of the Ras-ERK1/ERK2 pathway and suppressing tumor onset [84]. Interestingly, the regulatory effects of snoRNA-protein interactions on protein expression and activity have significant implications for DNA damage repair by binding to key proteins involved in this process, including DNA-dependent protein kinase (DNA-PK) catalytic subunit (DNA-PKcs) and poly (ADP-ribose) polymerases-1 (PARP-1). This binding allows snoRNAs to regulate their PTMs, such as phosphorylation and poly-adenosine diphosphate (ADP)-ribosylation (PARylation), and subsequently control the formation of protein complexes that participate in the DNA damage repair [15, 18, 22] (Fig. 2e). Although evidence of snoRNA-DNA repair protein interaction is limited at present, available research suggests that this pathway plays a crucial role in mediating the functions of snoRNAs in DDR. Detailed discussions on this mechanism can be found in the following sections.

## snoRNAs in DDR

**Fig. 3:**
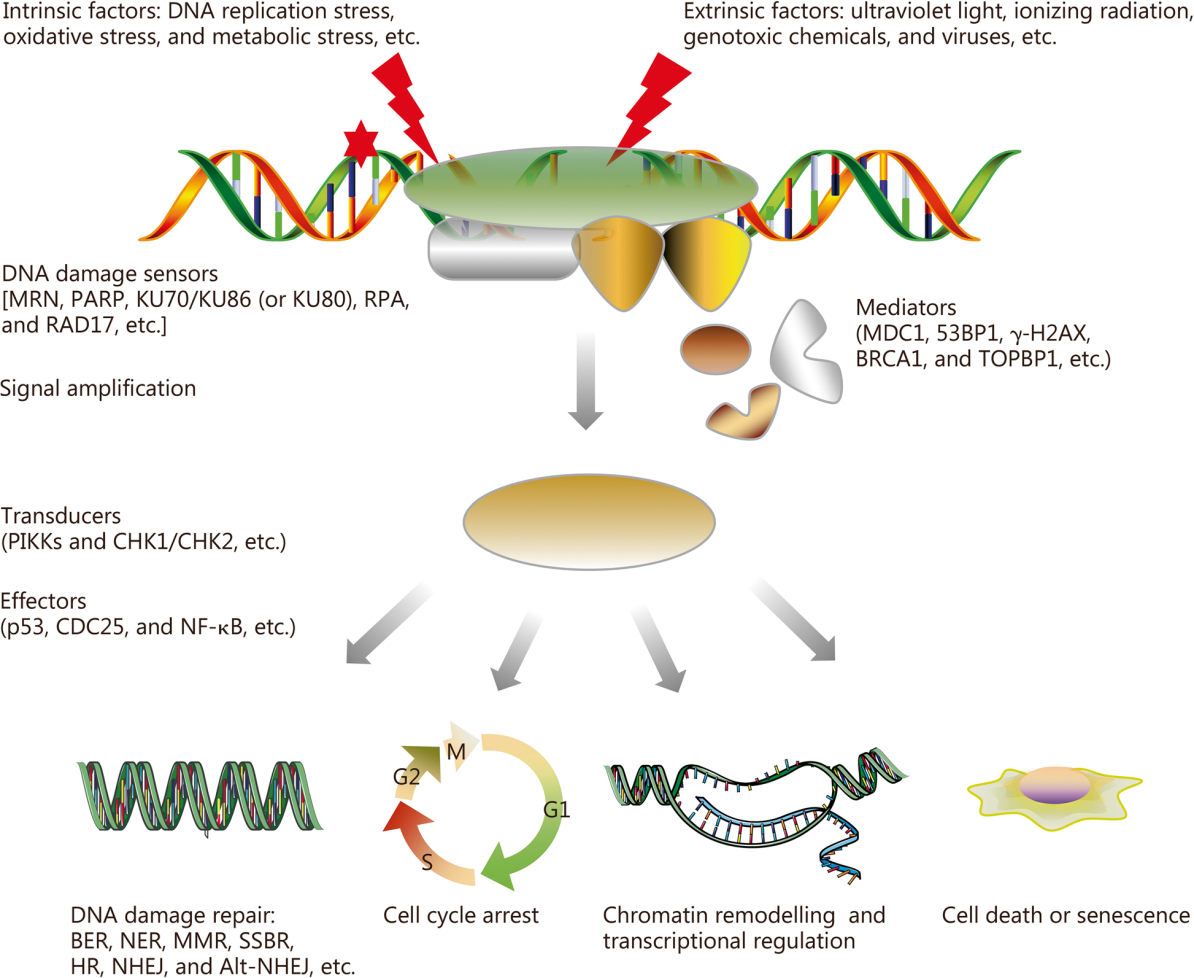
Primary process of DNA damage response (DDR). PIKK phosphatidylinositol 3-kinase-related kinase, MRN MRE11-RAD50-NBS1, PARP poly adenosine diphosphate (ADP)-ribose polymerases, KU KU70/KU86 (or KU80) heterodimer, RPA replication protein A, RAD17 radiation sensitive 17, MDC1 mediator of DNA damage checkpoint 1, 53BP1 p53-binding protein 1, γ-H2AX γ-H2A variant X, BRCA1 breast cancer susceptibility protein 1, TOPBP1 DNA topoisomerase II-binding protein 1, CHK checkpoint kinase, CDC cell division cyclin, NF-κB nuclear factor-κB, BER base excision repair, NER nucleotide excision repair, MMR mismatch repair, SSBR single-strand break repair, HR homologous recombination, NHEJ nonhomologous end joining, Alt-NHEJ alternative-NHEJ

Shortly after the discovery of the double helix structure of the DNA molecule by Watson et al. [85], it was found that the homeostasis of DNA molecules is regulated by a series of biochemical reactions inside the cell. A range of endogenous and exogenous stress factors rapidly activate the DDR system in cells, which uses DNA damage sensors to identify changes in chromatin structure. Subsequently, it recruits various DNA repair molecules to the damaged sites, initiating a signal cascade response. Activation of DNA damage checkpoint kinase and cell cycle checkpoint kinase triggers DNA damage repair and cell cycle blockade. These processes induce beneficial alterations in the transcriptional spectrum necessary for maintaining genome stability or inducing immune response, and inflammation, as well as various forms of cell death to eliminate severely damaged cells that are difficult to repair (Fig. 3). It has recently been shown that snoRNAs are involved in regulating DDR processes such as oxidative stress response, DNA damage repair, cell cycle regulation, and cell death [16, 22, 86–89]. Moreover, ionizing radiation-induced DDR significantly affects the expression of several snoRNA molecules [87, 90]. However, there is currently no comprehensive scientific theory explaining clearly the role of snoRNAs in DDR.

### snoRNAs in oxidative stress response

Cells generate ROS, such as superoxide, hydrogen peroxide (H_2_O_2_), peroxide, and hydroxyl-free radicals, through physiological and biochemical processes including respiration and energy metabolism. Additionally, cells produce reactive nitrogen species (RNS) like nitric oxide free radical and peroxynitrite. An increase in the generation of ROS and RNS disrupts the oxidation/reduction system balance, leading to oxidative stress. Physiological levels of ROS are very important in the regulation of physiological processes, but excessive oxidative stress products can cause damage to large biological molecules, including nucleic acids, proteins, and lipids. In particular, oxidative damage to DNA molecules is known to contribute to a diverse range of diseases, such as cardiovascular disease, cancer, aging, and inflammation [91, 92]. One prominent marker of DNA oxidative damage is the 8-hydroxydeoxyguanosine adduct which alters the spatial structure of the double-strand DNA leading to base mutations as well as SSBs, and DSBs.

**Fig. 4:**
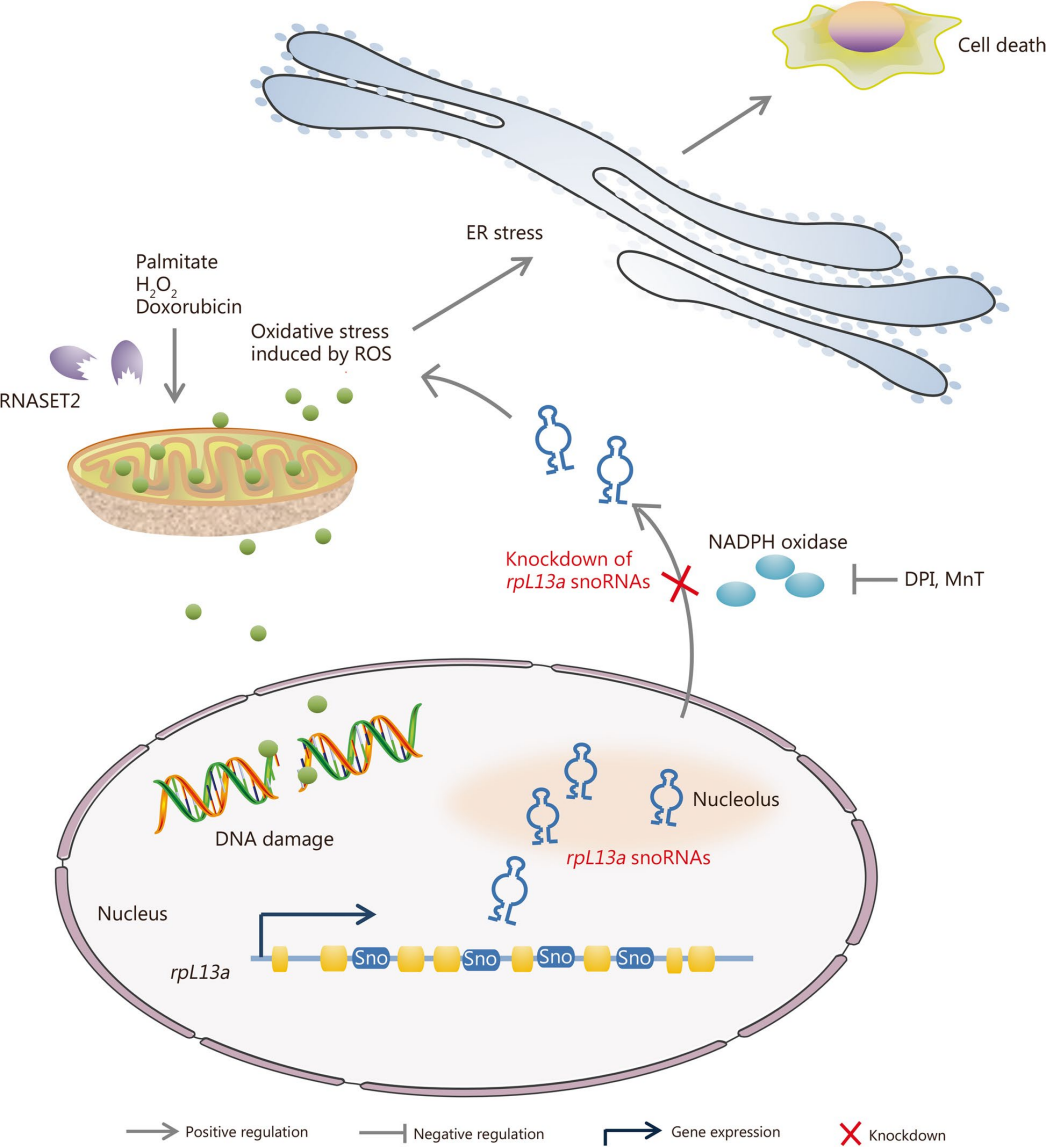
Expression and localization of small nucleolar RNAs (snoRNAs) in response to oxidative stress. For example, *rpL13a* snoRNAs shuttle from the nucleolus to the cytoplasm during the oxidative stress response, depending on the function of NADPH oxidase. The translocation of these snoRNAs induces endoplasmic reticulum (ER) stress-associated cell death. Sno snoRNA, rpL13a ribosomal protein L13a, DPI diphenyleneiodonium chloride, MnT Mn (III)TMPyP, NADPH nicotinamide adenine dinucleotide phosphate, H_2_O_2_ hydrogen peroxide, ROS reactive oxygen species, RNASET2 ribonuclease T2

Researchers have identified a limited number of snoRNAs that exhibit expression or localization changes under conditions of oxidative stress and subsequently play a role in regulating the oxidation/reduction balance. *U32a*, *U33*, and *U35a* are C/D box snoRNAs encoded by the 2nd, 4th, and 6th intron regions of the gene encoding (*rpL13a*). These particular snoRNAs are collectively known as “*rpL13a* snoRNAs” (Fig. 4). In 2011, Michel et al. [16] discovered that oxidative stress responses induced by palmitic acid and H_2_O_2_ significantly increase the expression of *U32a*, *U33*, and *U35a* in the cytoplasm without affecting their expression in the nucleolus. Similarly, in mice with lipid metabolism abnormalities induced by oxidative stress, there is an upregulation of *U32a*, *U33*, and *U35a* expressions in the liver. Knockdown experiments conducted in vitro revealed that these snoRNAs enhance cell resistance to oxidative stress induced by lipotoxic drugs and H_2_O_2_ while inhibiting the propagation of oxidative stress response in vivo. Of note, the underlying process does not rely on the 2’-O-Me modification of rRNA mediated by snoRNAs. Subsequently, Holley et al. [86] from the same research team provided further confirmation that DNA-damaging drug doxorubicin-induced oxidative stress response alters the distribution of *rpL13a* snoRNAs (*U32A*, *U33*, and *U3*4) in the nucleus/cytoplasm through a pathway dependent on superoxide and nicotinamide adenine dinucleotide phosphate (NADPH) oxidase, resulting ultimately in their rapid accumulation in the cytoplasm. Additionally, Caputa et al. [93] associated the aforementioned process with the regulation of ROS by the ribonuclease T2 (RNASET2) (Fig. 4). RNAomics data indicate that the effect of doxorubicin-induced oxidative stress exerts a universal influence on the expression and distribution of snoRNAs. It is noteworthy that this process may have a more significant impact on the nuclear/cytoplasm distributions for C/D box snoRNAs than H/ACA box snoRNAs and scaRNAs [86].

It is important to note that alterations in snoRNA expression and localization are not a prerequisite for their involvement in oxidative stress responses. Chu et al. [94] identified an orphan snoRNA, *ACA11* (*SCARNA22*), encoded by an intron of *WHSC1*, which is aberrantly expressed at high levels in multiple myeloma. Importantly, *ACA11* reduces ROS levels induced by H_2_O_2_ in mouse fibroblasts and enhances the resistance of multiple myeloma cells to chemotherapeutic drugs. Sletten et al. [95] confirmed that *SNORA73* regulates oxidative stress response induced by lipid metabolism both in vitro and in vivo. The knockdown of *SNORA73* expression increases the levels of antioxidant factors such as glutathione (GSH), NADPH, and nicotinamide adenine dinucleotide (NADH), thereby enhancing cellular tolerance to oxidative stress and lipotoxic drugs. However, neither of these studies identified any changes in snoRNA expression or localization. In summary, although there is evidence to suggest that snoRNAs play a prominent role in oxidative stress responses, their mechanism of action remains largely unknown.

### snoRNAs in DNA damage repair

Several DNA damage repair mechanisms exist within the cell, including direct repair (such as photoreactivation), translesion synthesis, base excision repair (BER), nucleotide excision repair (NER), mismatch repair (MMR), SSB repair, and DSB repair pathways. DSB repair pathways encompass homologous recombination (HR), nonhomologous end joining (NHEJ), and microhomology-mediated end joining (MMEJ; also known as alternative-NHEJ, Alt-NHEJ). These distinct responses are responsible for addressing various types of damage to maintain the integrity of DNA molecules and genetic stability. SSBs and DSBs represent two common forms of DNA damage. Typically, ionizing radiation-induced DNA damage events result in a higher number of SSBs compared to DSBs. However, DSBs pose a greater threat to the cellular viability of SSBs [2, 96]. PARP-1 and PARP-2 recognize SSBs and recruit the X-ray cross-complementing protein complexes to facilitate single-strand end-joining [97]. The meiotic recombination 11 (MRE11)-radiation sensitive 50 (RAD50)-Nijmegen breakage syndrome 1 (NBS1) (MRN) complex comprising MRN rapidly detects DSB sites and recruits/activates phosphatidylinositol 3-kinase-related kinases (PIKKs) during the early stages of DDR, along with their downstream substrate molecules such as γ-H2A variant X (γ-H2AX), mediator of DNA damage checkpoint 1 (MDC1), p53-binding protein 1 (53BP1), breast cancer susceptibility protein 1 (BRCA1), and p53 to initiate the DSB repair pathways [98].

#### snoRNA regulation of PARP-1 expression and activity

**Fig. 5:**
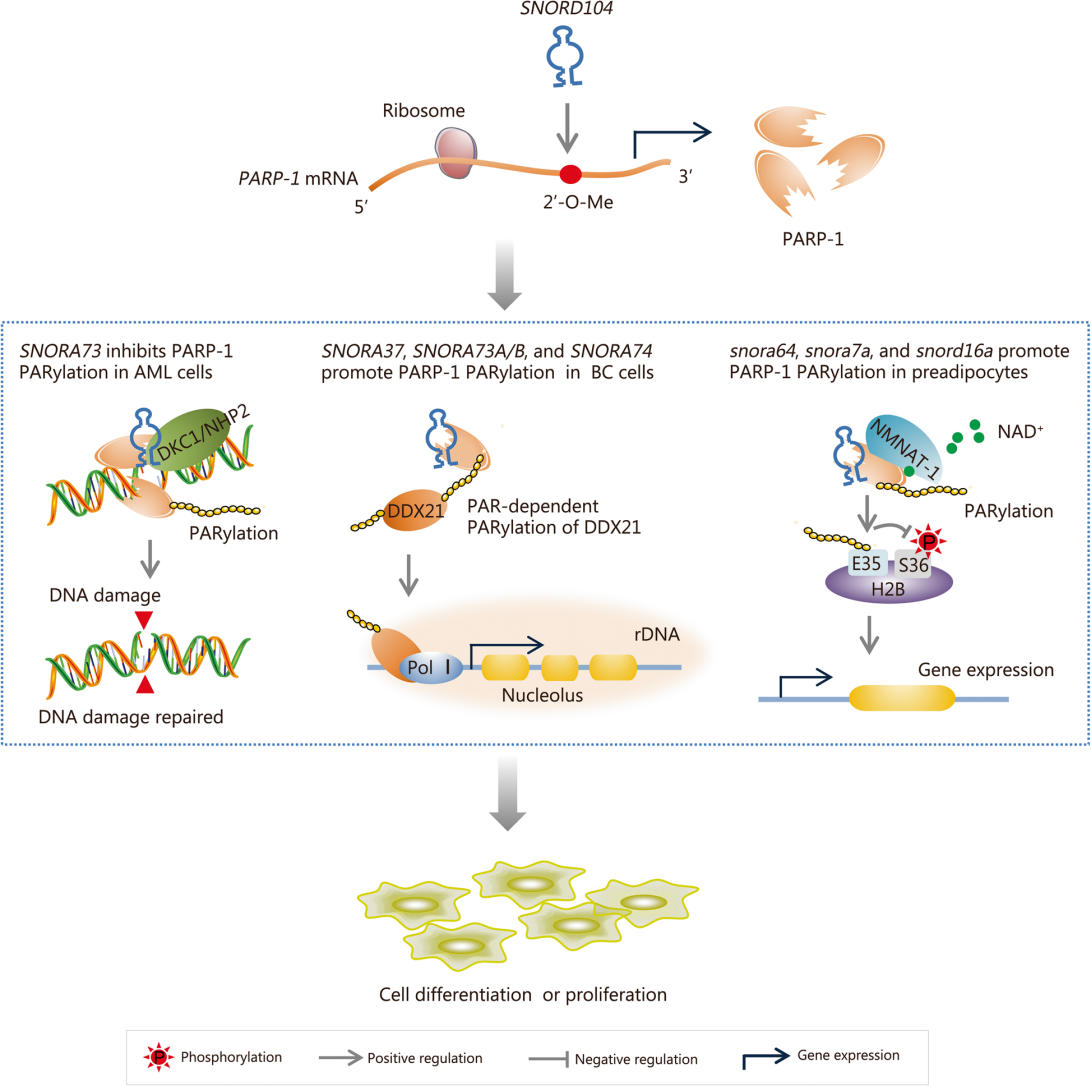
Small nucleolar RNAs (snoRNAs) regulation of PARP-1 expression and activity. C/D box *SNORD104* enhances the stability of *PARP-1* mRNA through 2’-O-methylation (2’-O-Me), thereby promoting the expression of PARP-1 protein. *SNORA73* (human), *SNORA37* (human), *SNORA74* (human), *snora64* (mouse), *snora7a* (mouse), and *snord16a* (mouse) are snoRNAs involved in PARP-1-mediated PARylation, exerting either positive or negative effects on cell differentiation and proliferation. SNORD box C/D small nucleolar RNA, PARP-1 poly adenosine diphosphate (ADP)-ribose polymerases-1, PARylation poly-ADP-ribosylation, AML acute myeloid leukemia, DKC1 dyskerin pseudouridine synthase synthase 1, NHP2 H/ACA ribonucleoprotein complex subunit 2, BC breast cancer, DDX21 DExD-box helicase 21, PAR poly(ADP-ribose), rDNA ribosomal DNA, NMNAT-1 nicotinamide mononucleotide adenylyltransferase-1

PARP-1 is a crucial sensor that plays a pivotal role in the initial stages of DDR. It primarily regulates DNA-protein interactions during the DNA repair processes for SSBs and DSBs by catalyzing PARylation of itself (autoPARylation) as well as substrate molecules. Undoubtedly, PARP-1 has emerged as an indispensable target for clinical antitumor therapy [99]. However, the precise molecular mechanisms underlying the PARylation of PARP-1 and its regulation of DNA damage repair remain incompletely elucidated. Recent discoveries have unveiled the involvement of a limited number of snoRNAs in mediating PARylation modification by PARP-1 or regulating post-transcription modification of *PARP-1* mRNA. These findings have provided novel insights into revealing the biological functions of PARP-1 and have garnered significant attention from researchers in the field of DNA damage (Fig. 5). In 2019, Kim et al. [100] conducted a screening and identification of snoRNAs that interact with PARP-1. In vitro experiments revealed direct binding of several snoRNAs (including *SNORA37*, *SNORA73A*, *SNORA73B*, and *SNORA74A*) to the DNA binding domain of PARP-1, ultimately leading to an increase in its PARylation levels. Additionally, the snoRNA-mediated autoPARylation of PARP-1 enhances its effect on DEAD (Glu-Asp-Ala-Glu) box RNA helicase 21 (DDX21) and activates the transcription of rDNA. Subsequently, Huang et al. [101] reported that *snora64*, *snora7a*, and *snord16a* activate PARP-1 in mouse cells by promoting autoPARylation, which increases PARylation while inhibiting phosphorylation of H2B protein, facilitating the transcription of genes that may be involved in DDR. Therefore, does the snoRNA-mediated regulation of PARylation have a significant impact on the DNA damage repair pathway mediated by PARP-1? The research performed by Han et al. [18] in 2022 provided more powerful evidence for this question. Indeed, they found that DNA damage stress reduces the affinity between *SNORA73* and chromatin. Through a stem-loop structure at the 5’ end that is rich in GC sequences, *SNORA73* negatively regulates both the PARylation level and the catalytic activity of PARP-1. The inhibition of *SNORA73* expression reduces the levels of DNA damage in acute myeloid leukemia (AML) cells while enhancing genome stability. However, it should be noted that there are inconsistencies regarding the regulation of PARylation mediated by *SNORA73* across different research models; currently, unclear reasons account for these discrepancies [18, 100]. Lu et al. [102] recently revealed a positive correlation between C/D box *SNORD104* and the expression of *PARP-1* in endometrial cancer cells. *SNORD104* indirectly increases the stability of *PARP-1* mRNA by regulating 2’-O-Me, indicating that snoRNAs are involved in the expression of the *PARP-1* gene at the transcriptional level. This is sufficient evidence to demonstrate the complexity of snoRNA regulation of DNA repair factors (e.g., PARP-1), and that in some cases, snoRNAs may act synchronously at multiple levels.

#### snoRNA regulation of the activity of proteins in the PIKK family

In the PIKK family, there are three serine/threonine-directed kinases involved in DDR: ataxia telangiectasia mutated (ATM), ATM and Rad3-related (ATR), and DNA-PKcs. Additionally, the PIKK family includes mammalian target of rapamycin (mTOR), suppressor of morphogenesis in genitalia 1 (SMG1), and transformation/transcription domain-associated protein (TRRAP). These DDR-related PIKKs exhibit homology with conserved domains, including the PI3K kinase domain, FRAP-ATM-TRRAP (FAT) domain, the FAT C-terminal (FATC) domain, and Huntingtin, elongation factor 3, protein phosphatase 2A, and TOR1 (HEAT) repeat domain. They phosphorylate a wide range of substrate proteins on the SQ/TQ sequence, sharing numerous substrates and establishing close interactions with each other [103–105].

DNA-PK is a complex consisting of a large catalytic subunit DNA-PKcs and a heterodimer protein composed of KU70/KU86 (or KU80 in mice), which binds to damaged DNA ends. This complex plays a crucial role in regulating the processing of DSB in the early stages of DNA damage repair. It recruits various repair molecules, including DNA ligase I, X-ray repair cross-complementing protein 4 (XRCC4), XRCC4-like factor, and Artemis to the damaged site for efficient completion of DNA end joining through the NHEJ pathway [103, 106]. The large catalytic subunit, DNA-PKcs, is composed of 4128 amino acids and normally adopts a conformation that inhibits its kinase activity by blocking substrate binding sites. However, upon the occurrence of DSBs, KU70/KU86 (or KU80) rapidly recognizes the broken ends and initiates the NHEJ repair process. Then, DNA-PKcs interacts with KU70/KU86 (or KU80), inducing a conformational change that facilitates autophosphorylation at the Ser2056 (S2023 – S2056) and Thr2609 (T2609 – T2647) sites followed by activation [107]. Hence, Ser2056 and Thr2609 phosphorylation levels are considered the hallmarks reflecting the activity status of DNA-PKcs. Finally, activated DNA-PKcs phosphorylates specific serine residues on extensive downstream substrates.

**Fig. 6:**
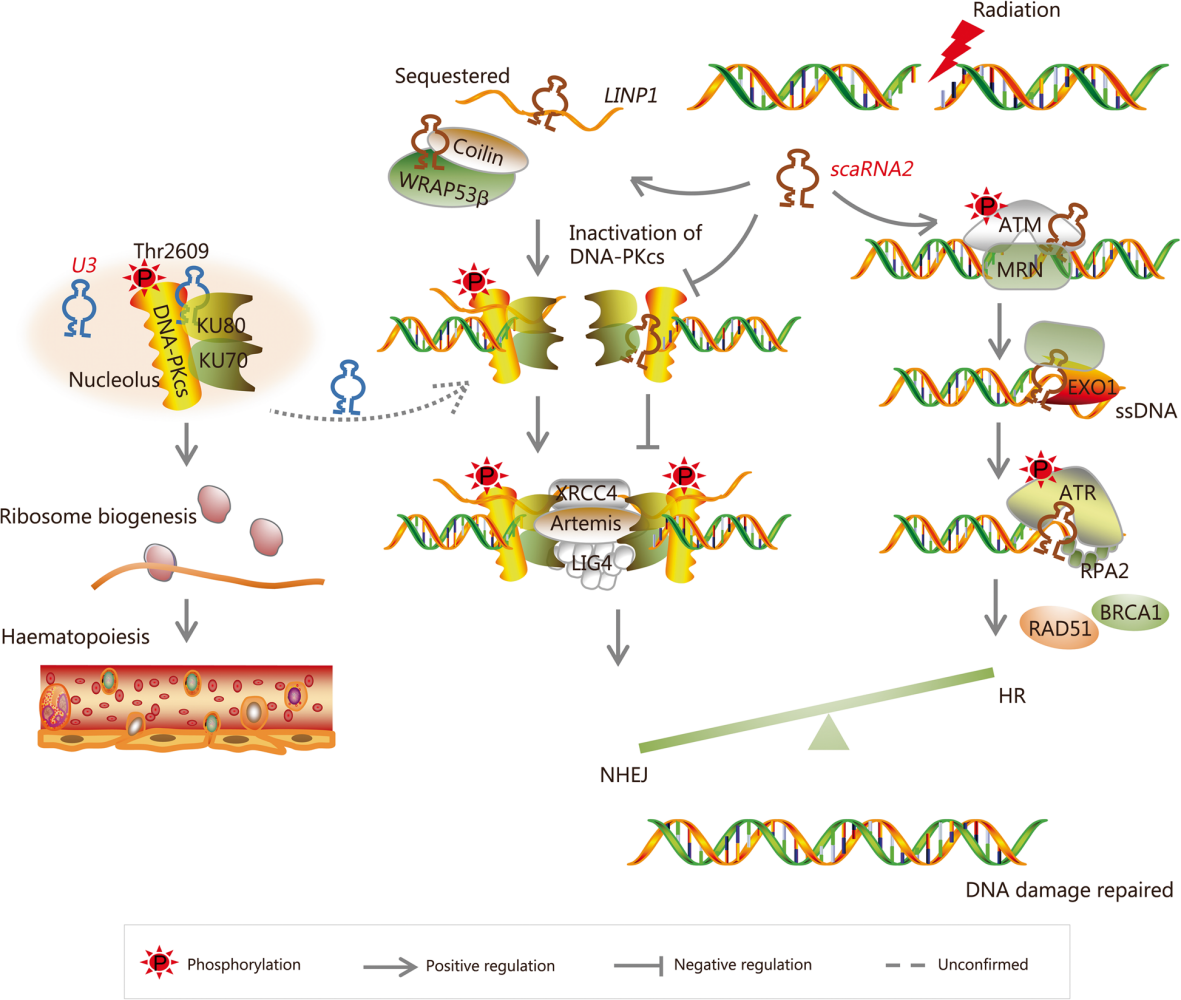
Small nucleolar RNAs (snoRNAs) regulation of the activity of DDR-related PIKKs. DNA-PKcs is phosphorylated at the Thr2609 site by *U3* during hematopoiesis in mice. However, the involvement of *U3*-mediated activation of DNA-PKcs in DDR remains unknown. *scaRNA2* appears as a negative regulator of DNA-PKcs activation. It binds to DNA-PKcs and weakens its interaction with KU70/80 subunits, thereby inhibiting the autophosphorylation of DNA-PKcs at the Ser2056 and Thr2609 sites. Meanwhile, *scaRNA2* sequesters *LINP1* to inhibit the activity of DNA-PKcs. The obstruction of DNA-PKcs activation induced by *scaRNA2* prompts cells to opt for HR repair in DDR, and undoubtedly, the *scaRNA2*-mediated phosphorylation of ATR contributes to this process. *U3* small nucleolar RNA U3, LINP1 lncRNA in nonhomologous end joining pathway 1, scaRNA small Cajal body-specific RNA, DNA-PKcs DNA-dependent protein kinase catalytic subunit, ATM ataxia telangiectasia mutated, MRN MRE11-RAD50-NBS1, XRCC4 X-ray repair cross-complementing protein 4, LIG4 DNA ligase 4, EXO1 exonuclease 1, ssDNA single-stranded DNA, ATR ATM and Rad3-related, RPA2 replication protein A2, BRCA1 breast cancer susceptibility protein 1, RAD51 radiation sensitive 51, HR homologous recombination, NHEJ nonhomologous end joining

The activity of DNA-PKcs is crucial for maintaining the efficiency of normal NHEJ repair. Consequently, a reduction in cellular DNA-PKcs activity leads to impaired DNA damage repair, whereas an abnormal increase in activity inhibits HR repair through a competitive selection between NHEJ and HR repair mechanisms, ultimately elevating the risk of erroneous DNA repair [108]. The known regulatory factors of DNA-PKcs include proteins such as KU70/KU86 (or KU80), ATM, and ATR. Although ncRNAs, including lncRNAs (e.g., *LINP1*) and miRNAs (e.g., *miR-101*), have been identified as regulators of DNA-PKcs, an interaction between snoRNAs and DNA-PKcs was only discovered in the last 2 – 3 years [109, 110]. In 2020, Shao et al. [15] published a study in *Nature* demonstrating that the 5’ end stem-loop structure of snoRNA *U3* directly binds to both DNA-PKcs and KU86 in the nucleolus. Moreover, this interaction promotes the phosphorylation of DNA-PKcs at the Thr2609 site without activating the Ser2056 site, ultimately resulting in the phosphorylation of the p53 substrate protein. Furthermore, the study provided evidence that *U3*-mediated DNA-PKcs phosphorylation at Thr2609 promotes mouse bone marrow hematopoiesis by regulating 18S rRNA production. Although the authors stated that this biological function does not depend on the classic NHEJ repair mechanism, it presented initial evidence for direct regulation of DNA-PKcs activity and function by snoRNAs (Fig. 6). Unexpectedly, it also broadened the understanding by linking the role of DNA-PKcs from NHEJ repair to rRNA processing and hematopoiesis. Subsequently, Bergstrand et al. [22] provided further evidence that snoRNAs regulate the activity of DNA-PKcs in the process of DNA damage repair. The authors discovered that *scaRNA2* binds to DNA-PKcs through its C/D box in human breast cancer cells and osteosarcoma cells, thereby preventing the forming of a complete DNA-PK complex with KU protein and inhibiting autophosphorylation at Ser2056 and Thr2609 sites. Furthermore, *scaRNA2* competitively binds with lncRNA *LINP1* to inhibit the activity of DNA-PKcs. Hence, their study provided evidence suggesting that *scaRNA2* promotes the recruitment of multiple HR repair factors at DSB sites by negatively regulating DNA-PK activity and inhibiting NHEJ repair (Fig. 6). Moreover, the snoRNA-hosting lncRNA molecule *SNHG12* acts as a molecular scaffold that interacts with DNA-PKcs, positively regulating the interaction between DNA-PKcs and KU70/KU80 and subsequently promoting NHEJ repair [111]. Therefore, focusing on the role of host genes in DNA repair may contribute to exploring the functions of snoRNAs owing to their ambiguous interaction with host genes.

Similar to DNA-PKcs, ATM specifically regulates DSB repair. The MRN complex recruits ATM to the damage site, where it undergoes activation through autophosphorylation at Ser1981 and then phosphorylates various DNA repair molecules and the cell cycle checkpoint kinases to mediate a signal cascade response [103, 104]. Patients with ataxia-telangiectasia (A-T) syndrome caused by ATM mutations typically exhibit heightened radiosensitivity and an increased propensity for cancer development [6]. Bergstrand et al. [22] found that *scaRNA2* suppresses the catalytic activity of DNA-PKcs while promoting the recruitment and activation of ATM-mediated by MRN (Fig. 6). However, no studies have demonstrated the direct interaction between snoRNAs and ATM.

An important difference among DNA-PKcs, ATM, and ATR lies in the fact that the first two proteins primarily recognize double-stranded DNA (dsDNA) to enhance DSB repair, whereas ATR primarily senses single-stranded DNA (ssDNA) formed during DSB processing or DNA replication [112, 113]. ATR stably binds to DNA damage sites, undergoes autophosphorylation, and subsequently phosphorylates its substrate molecules to participate in the repair of various types of DNA damage such as DSBs, SSBs, crosslinks, and base adducts [103, 112, 114]. Notably, ATR is the most important kinase in response to DNA replication stress, and it is of great significance for DNA damage repair during proliferation, faithfully maintaining DNA replication and cell survival. The cofactors ATR-interacting protein (ATRIP), DNA topoisomerase II-binding protein 1 (TOPBP1), and Ewing’s tumor-associated antigen 1 promote the recruitment and activation of ATR at DNA damage sites [103, 114], with mutations or deletions in *TOPBP1* having lethal effects on mammalian cells [115, 116]. Researchers have proposed that DSB damage occurring during the replication process (S-phase) mainly relies on HR repair [104]. At the initial stage of DSB resection, the ATR-ATRIP complex senses and binds to the replication protein A (RPA)-coated 3’ ssDNA overhangs produced by the endonucleases MRE11 and exonuclease 1 (EXO1). This leads to phosphorylation of C-terminal binding protein interacting protein (CtIP) which promotes HR repair [113]. In a recent study published by Chen et al. [87], it was reported that the snoRNA *scaRNA2* regulates ATR phosphorylation during radiation-induced DSB repair. They found that *scaRNA2* expression is regulated by ATR and ATM, and it is upregulated following ionizing radiation exposure. *scaRNA2* binds to ATR protein at the 3’ end, thereby modulating MRE11 and EXO1-mediated DSB resection process leading to enhanced activation of ATR (Fig. 6). Knockdown experiments targeting *scaRNA2* resulted in suppressed HR repair efficiency along with increased radiation-induced DSBs, and improved the sensitivity observed specifically colorectal cancer cells when subjected to radiotherapy. To date, this represents the sole study elucidating the regulation of ATR function by snoRNA.

In summary, research on the snoRNA-mediated regulation of PIKKs has the potential to significantly enhance our comprehension of their biological functions. This may help elucidate the physiological and pathological significance of PIKKs from multiple perspectives and enable the development of more efficacious clinical treatment strategies.

#### snoRNA regulation of p53 expression and activity

**Fig. 7:**
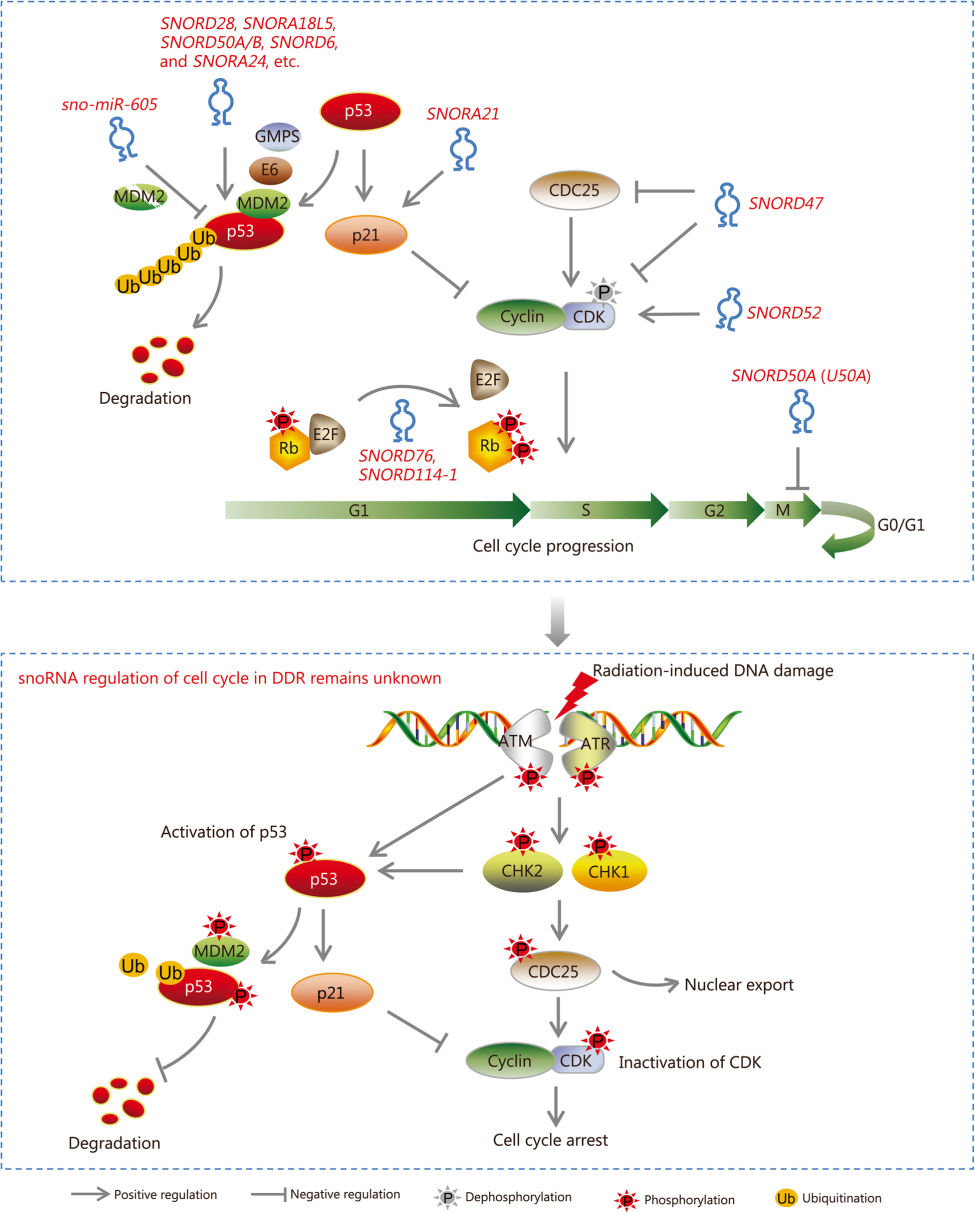
Small nucleolar RNAs (snoRNAs) regulation of cell cycle checkpoints. The cyclin/CDK complex refers to cyclinD-CDK4/6, cyclinE/A-CDK2, or cyclinB1/CDK1. The G1/S checkpoint is primarily regulated by cyclinD-CDK4/6 and cyclinE/A-CDK2 complexes via the phosphatase CDC25-mediated dephosphorylation, thereby facilitating the G1/S transition. Meanwhile, p53 and p21 act as inhibitors during the transition, whereas the phosphorylated Rb acts as an activator. Several snoRNAs participate in G1/S transition by regulating the expression of p53, p21, and Rb, as depicted in the figure. Likewise, the cyclinB1/CDK1 complex, activated by CDC25, governs the activity of the G2/M checkpoint to mediate the transition from the G2 phase to the M phase. *SNORD47* and *SNORD52* are involved in G2/M transition by targeting either CDC25 or cyclinB1/CDK1. In addition, *U50A* acts as an inhibitor in mitosis. In DDR (e.g., ionizing radiation-induced DNA damage), CDC25 is inactivated by ATM-CHK2/ATR-CHK1-mediated phosphorylation, ultimately leading to the inactivation of CDKs and causing cell cycle arrest. The snoRNA-mediated regulation of the cell cycle in DDR remains unknown. SNORD box C/D small nucleolar RNA, SNORA box H/ACA small nucleolar RNA, MDM2 mouse double minute 2, E6 human papillomavirus oncoprotein E6, GMPS guanosine 5’-monophosphate synthase, CDC25 cell division cyclin, CDKs cyclin-dependent kinases, E2F E2 family, Rb retinoblastoma tumor suppressor protein, DDR DNA damage response, CHK checkpoint kinase, ATM ataxia telangiectasia mutated, ATR ATM and Rad3-related, Ub ubiquitination

The p53 protein, encoded by *TP53*, functions as a multifunctional nuclear transcription factor maintaining cellular homeostasis during various stress response states, including stress responses to DNA damage, oxidative stress, and metabolic stress. It also prevents uncontrolled cell proliferation after exposure to these stressors. However, the wild-type p53 protein is extremely unstable in cells. Its stability is significantly influenced by PTMs such as ubiquitination, phosphorylation, and acetylation. Notably, the p53 expression under normal conditions is negatively regulated by E3 ubiquitin-protein ligase mouse double minute 2 (MDM2) through a p53-MDM2 feedback loop. Activation of p53 promotes the expression of the *MDM2* gene and subsequent binding of MDM2 protein to the N-terminus of p53, resulting in its ubiquitination, ultimately facilitating the 26S proteasome-mediated degradation [117, 118]. During the early stages of DDR, activated DNA damage checkpoint proteins, including ATM/ATR, DNA-PKcs, and checkpoint kinase (CHK)1/CHK2, phosphorylate the Ser15 and Ser20 sites on p53, thereby inhibiting the MDM2-mediated degradation (Fig. 7). Conversely, HIV-1 tat-interacting protein 60 kD (TIP60) and human males absent on the first (hMOF) regulate the acetylation of p53 protein that promotes its binding to DNA while inhibiting the interaction between p53 and MDM2 [119, 120]. The accumulation of p53 protein promptly activates downstream signaling pathways that promote various DNA damage repair mechanisms (including NER, BER, MMR, NHEJ, and HR), induce cell cycle arrest, and promote apoptosis via transcriptional regulation [119]. In general, mutations in *TP53* and the disturbed regulation of p53 protein stability are the most critical causes of its dysfunction.

More studies have been published on the snoRNA-mediated regulation of the p53 pathway compared to PARP-1 and DNA-PKcs (Fig. 7). Following the discovery of a “parent-child relationship” between snoRNAs and miRNAs [74], Xiao et al. [75] found that *sno-miR-605* binds to the 3’ UTR region of *MDM2* mRNA, inhibiting its translation. Simultaneously, the activated p53 increases the expression of *sno-miR-605*, producing a p53-*sno-miR-605*-MDM2 positive feedback loop that promotes cell apoptosis. Thus, this study established a connection between snoRNAs and the p53 pathway for the first time. Additionally, other researchers reported that the expression of snoRNA *U44* and *U47* is induced by DNA damage in cells treated with adriamycin, and this process is dependent on wild-type p53 expression [121]. In 2015, Yu et al. [76] further explored the relationship between snoRNAs and the p53 pathway using a model with induced/activated p53 in cells. They found that the expression of several snoRNA molecules derived from *SNHG1* (specifically *SNORD22/25/26/27/28*) was suppressed by p53. Moreover, while *SNORD28* is processed into a small miRNA molecule called *sno-miR-28*, its expression is inhibited by p53 through the *SNHG1*-*sno-miR-28* axis. TATA-box binding protein associated factor 9b (TAF9B) acts as a known coactivator for p53 by competitively binding to MDM2 to regulate protein stability. However, Yu et al. [76] findings suggest that *sno-miR-28* negatively regulates *TAF9B* mRNA and protein expression via an intricate feedback loop involving *SNORD28*-*sno-miR-28*-TAF9B-p53 interactions which ultimately impact cell proliferation and survival. Although this study established the involvement of snoRNAs in regulating p53 protein expression, it does not provide definitive evidence regarding their influence of *SNORD28* (or *sno-miR-28*) on the stability of p53 protein.

In 2018 and 2021, two teams from China identified two types of snoRNAs involved in the regulation of p53 protein stability: *SNORA18L5* and *SNORD50A/B* [122, 123]. Cao et al. [122] found that the overexpression of *SNORA18L5* induces an active ribosome biogenesis response, leading to the sequestration of RPL5 and RPL11 in the nucleus and inhibiting their binding with MDM2. Consequently, this results in increased MDM2-mediated ubiquitination of p53 in the cytoplasm, thereby promoting p53 protein degradation and ultimately leading to the development of hepatocellular carcinoma (HCC). Apart from MDM2, tripartite motif-containing protein 21 (TRIM21) is an important regulator of p53 protein through its function as a ubiquitin E3 ligase. TRIM21 regulates the ubiquitination level of guanine 5’-monophosphate synthase (GMPS) protein, determining its nucleo-cytoplasmic distribution. In the cell nucleus, GMPS recruits ubiquitin-specific-processing protease 7 (USP7) to bind with p53 and form a complex that facilitates the deubiquitination of the p53 protein [124]. Su et al. [123] demonstrated that *SNORD50A/B* negatively regulates wild-type p53 protein expression. Moreover, *SNORD50A/B* knockout inhibits the TRIM21-mediated ubiquitination of GMPS, thus promoting the GMPS/USP7/p53 interaction, reducing p53 ubiquitination, while increasing its stability. Additionally, Li et al. [83] recently reported that *SNORD6* binds to the E6 protein in cervical cancer cells, enhancing the interaction between the E6/E6AP complex and p53 which ultimately promotes the ubiquitination and degradation of p53. Finally, the present research team revealed that the H/ACA box snoRNA *SNORA24* promotes p53 protein degradation through the proteasome pathway, inhibiting the activity of p53 in processes such as cell cycle, proliferation, and apoptosis and exerting an oncogenic function in colorectal cancer [125]. While scientists have provided significant insights into the modulation of p53 function by snoRNAs, there is still no firm evidence suggesting that snoRNAs modulate DDR through the p53 signaling pathway. It is speculated that the p53 network is an essential interface for linking snoRNAs to DDR.

### snoRNA regulation of cell cycle checkpoints

Cell cycle checkpoints, also known as “cell cycle restriction points”, include the G1/S phase, S phase, G2/M phase, and M phase checkpoints. These checkpoints are crucial defense mechanisms that enable cells to respond to DNA damage while providing the necessary time for DNA repair. Among these checkpoints, the G1/S and G2/M checkpoints are the most important for DDR. Activation of the G1/S checkpoint induces arrest at the G1 phase, facilitating the repair of damaged molecules before initiation of DNA replication and preventing replication stress and base mismatches. On the other hand, activation of the G2/M checkpoint results in arrest at the G2 phase, thereby impeding entry into mitosis by damaged cells. This serves as a critical barrier against chromosomal aberrations and protects cells from mitotic catastrophe. The normal cell cycle is mainly regulated by cell cycle proteins such as cyclins, cyclin-dependent kinases (CDKs), CDK inhibitors (CKIs), and cell division cyclin 25 (CDC25) protein phosphatase. Notably, it is through the formation of the cyclin/CDK protein complex that central control over the cell cycle checkpoint is exerted. Disruption of these checkpoint functions leads to significant defects in DNA damage repair and increases the sensitivity of individuals or cells to genotoxic agents, resulting in genomic instability and carcinogenesis [3, 4].

#### G1/S checkpoint

The core regulators of the G1/S checkpoint are the cyclinD-CDK4/6 and cyclinE/A-CDK2 complexes, whose functions are regulated by a combination of positive and negative regulatory mechanisms. The G1/S phase transition is regulated by the phosphorylation level of the Rb protein. The increased expression of cyclinD-CDK4/6 during the transition from the early to late G1 phase promotes the hyperphosphorylation of Rb, leading to the release and activation of E2F protein. Activated E2F acts as a core transcription factor that regulates the expression of cyclinE/A-CDK2 and DNA replication-related genes. Simultaneously, CDC25 dephosphorylates and activates CDKs, which further increase the phosphorylation level of Rb. This forms a positive feedback loop with E2F, promoting the G1/S phase transition [4, 98, 126]. Conversely, p53 and CKIs (such as p21 and p16) are the main inhibitory factors for cyclinD-CDK4/6 and cyclinE/A-CDK2. DSBs activate the ATM-CHK2 pathway while SSBs activate the ATR-CHK1 pathway respectively, resulting in the phosphorylation of p53 protein and promotion of p21 expression, thereby inducing G1/S phase arrest (Fig. 7).

In a study on leukemia, Valleron et al. [88] found that *SNORD114-1* [*14q*(*II-1*)] positively regulates the phosphorylation of Rb at the Ser780 site, thereby controlling the distribution of G1/S phase through the Rb/p16 pathway and impacting the proliferation and differentiation of leukemia cells. Similarly, *SNORD76* alters the distribution of the G1/S phase in glioblastoma cells and induces S phase arrest by modulating Rb protein expression and its phosphorylation level [127]. However, the precise mechanisms used by *SNORD114-1* and *SNORD76* to regulate Rb expression and activity are not clear. The p53 protein acts as a key inhibitor of the G1/S phase transition by regulating the expression of p21, as described in Fig. 7. Notably, studies on snoRNAs that influence the stability of p53 protein (e.g., *SNORA18L5*, *SNORD50A/B*, and *SNORA24*) have demonstrated significant alterations in the distribution of G1/S phase along with abnormal expression of G1/S checkpoint proteins [122, 123, 125]. Furthermore, the overexpression of *SNORA21* in gallbladder cancer cells inhibits cyclinD1 while increasing p21 expression, leading to G1 phase arrest and suppression of cell proliferation (Fig. 7) [128]. Although snoRNA *U3* has been shown to modulate G1/S phase arrest in human fibroblasts and gallbladder cancer cells (to a limited extent), the authors did not explain the underlying mechanism for this phenomenon [129].

#### G2/M checkpoint

The central component of the G2/M checkpoint is the cyclinB1/CDK1 protein complex. Wee1-like protein kinase (WEE1) and myelin transcription factor 1 (MYT1) phosphorylate CDK1 at its Tyr15 and Thr14 sites during the interphase cells, thereby leading to the inactivation of CDK1 kinase [126, 130, 131]. CDC25 (represented by the subtypes CDC25A, CDC25B, and CDC25C) is the most critical protein that directly activates CDK1. By dephosphorylating CDK1, CDC25 activates the cyclinB1/CDK1 complex, driving cells from the G2 phase to the M phase [132, 133]. Under DNA damage stress conditions, activated ATM and ATR phosphorylate CHK2 and CHK1. This, in turn, phosphorylates and inactivates CDC25, thereby inhibiting its dephosphorylation effect on CDK1 while activating the G2/M checkpoint.

As reported by Xu et al. [134] in 2017, the overexpression of the tumor suppressor snoRNA *SNORD47* in glioblastoma cells inhibits the expression of various G2/M checkpoint proteins, including cyclinB1, CDC25C, and CDK1, ultimately inducing G2 phase arrest. Li et al. [89] reported that the snoRNA *SNORD52*, which is negatively regulated by the tumor suppressor Up-frameshift 1, directly interacts with CDK1 in hepatoma cells to form an RNA-protein complex. Moreover, *SNORD52* regulates the ubiquitination and Thr161 phosphorylation levels of CDK1, enhancing CDK1 protein stability and activity to drive cells across the G2/M checkpoint and consequently inducing an oncogenic phenotype. Zhu et al. [135] found that *SNORA14A* induces G2/M phase arrest and cell apoptosis in hepatoma cells by regulating succinic acid metabolism, suggesting that *SNORA14A* is a potential diagnostic and prognostic marker of HCC. Additionally, snoRNA *U50A* (also known as *SNORD50A*) inhibits the expression of various mitosis-related genes (including *SMC5*, *ATRX*, *CENPE*, and *CENPF*) to delay mitosis. The overexpression of *U50A* induces M phase arrest in breast cancer cells, resulting in an anticancer effect [136]. In conclusion, the current research on snoRNAs regulating G2/M checkpoints is still limited, with only a few reports providing inadequate insights into the molecular mechanisms related to several snoRNAs (Fig. 7). Because of their significant impact on the activity of p53 and checkpoint kinases such as CDKs and CDC25C, snoRNAs are expected to become potential therapeutic targets for diseases like cancer.

### snoRNA involved in DNA damage-induced inflammation and immunity

Inflammation is a non-specific pathological response to local tissue injury, characterized by increased vascular permeabilization, edema, and leukocyte infiltration to the injured area. The relationship between inflammation and DNA damage has always been a hot topic for scientists. Inflammation induces intracellular oxidative stress reaction, leading to increased levels of ROS/RNS and various types of DNA damage. Conversely, DNA damage activates the transcription factors nuclear factor-κB (NF-κB) and interferon (IFN) regulatory factor 3 (IRF3), which trigger an inflammatory response through multiple pathways, promoting the expression of IFNs and other inflammatory factors [137, 138]. Effective DNA repair is crucial for alleviating the inflammatory response. In the case of defects in key DNA repair molecules such as bloom syndrome protein (BLM), Werner syndrome protein (WRN), ATM, and lamin A/C (LMNA) can lead to long-term chronic inflammation that disrupts the balance of the oxidation/reduction system and causes continuous accumulation of gene mutations, ultimately resulting in serious disorders such as cancer and senescence [137–141].

Immune-mediated inflammatory responses underlie the pathogenesis of various inflammatory diseases. Innate immunity, an innate and non-specific defense mechanism, is mediated by immune cells such as monocytes, macrophages, NK cells, mast cells, plasma cells, granulocytes, and antigen-presenting cells. Innate immunity plays an essential regulatory role in the process of DNA damage-induced inflammation. DNA damage leads to the accumulation of dsDNA (such as micronucleus) in the cytoplasm. The accumulation of dsDNA activates the DNA sensor cyclic guanosine monophosphate-adenosine monophosphate synthase (cGAS; with limited recognition capacity for ssDNA), subsequently promoting IFN-I expression through the cGAS-stimulator of interferon genes (STING)-IRF3 pathway, thereby activating the innate immune response signaling pathway to increase the expression of inflammatory factors [138, 142]. Besides recognizing dsDNA molecules from self-cells, cGAS can also sense exogenous bacterial or viral DNA or RNA (e.g., SRAS-CoV-2) [143, 144]. Various negative regulatory mechanisms exist to prevent the abnormal activation of the cGAS-STING pathway. The nuclease three-prime repair exonuclease 1 (TREX1) facilitates the removal of DNA fragments in the cytoplasm to prevent dsDNA accumulation. Dysregulation in nucleic acid metabolism leads to severe immune diseases such as Aicardi Goutières syndrome [145, 146]. The DNA repair regulator DNA-PKcs directly phosphorylates various substrates, including cGAS, IFI16, and IRF3, to inhibit the activity of the cGAS-STING pathway. Mutations in *PRKDC* (encoding DNA-PKcs) that lead to a functional deficiency in DNA-PKcs promote inflammatory responses in mouse and human cells [147]. CDK1-mediated phosphorylation at the Ser305 site of cGAS in mitotic cells inhibits the recognition of self-DNA by cGAS [148], while autophagy protein unc-51-like autophagy-activating kinase 1 (ULK1) blocks immune signaling by phosphorylating the STING protein [149]. However, dsDNA triggers innate immunity not only in this manner alone. Dunphy et al. [150] discovered that PARP-1 and ATM identify dsDNA induced by etoposide and rapidly activate NF-κB in a cGAS-independent manner, thereby activating native immune responses and inducing inflammation.

Adaptive immunity serves as the second line of defense in the immune system, involving the proliferation and differentiation of activated lymphocytes (primarily T cells and B cells) into effector cells through antigen stimulation. This is followed by a specific immune response mediated by antigen-antibody interactions. The DDR system, in addition to regulating innate immunity, also influences adaptive immunity by participating in the development of T and B cells. Variable (diversity) joining [V(D)J] recombination and class switch recombination (CSR) are critical stages in the early development of lymphocytes, determining the expression and diversity of antigen receptors (the immunoglobulin on the surface of B and T cell receptors). Under normal conditions, DSBs generated during this process are mainly repaired through the NHEJ pathway. Several DNA repair molecules, including ATM, DNA-PKcs, DNA ligase IV, and KU70/KU80, regulate V(D)J recombination and CSR [151, 152]. Studies have reported that ATM-deficient mice exhibit disorders in T cell development with reduced mature lymphocyte number [153–155]. More seriously, the dysfunction of NHEJ repair caused by DNA-PKcs mutations leads to the absence of mature T lymphocytes and B lymphocytes, resulting in severe combined immunodeficiency in mammals [156, 157].

Clinical studies have revealed that the expression of snoRNAs undergoes significant changes in certain diseases related to inflammation, autoimmune deficiency, and infection. Some of these snoRNAs participate in the activation, proliferation, and differentiation of immune cells and act as important regulators in innate and adaptive immune responses [158–162]. The inflammatory response induced by lipopolysaccharide stimulates the activation of macrophages and subsequent secretion of various snoRNA molecules encoded by *rpL13a*. These snoRNAs participate in vesicle-mediated cell-cell communication while also regulating the inflammatory response [163]. Zhang et al. [164] showed that *SNORD46* regulates the activity of NK cells and highlighted how *SNORD46* inhibitors can counteract immune tolerance among obese patients, thereby presenting promising prospects for antitumor immune technology based on CAT-NK cells. Several researchers have reviewed immune response-related snoRNAs and identified their critical involvement in the proliferation and activation of diverse immune cells, including T cells, B cells, macrophages, and dendritic cells [20, 163–165]. However, the biological mechanisms underlying these molecules remain unclear. In 2021, Wan et al. [166] revealed that translation stress along with collided ribosomes promotes DNA-dependent cGAS activation. As an essential component involved in the process of translation, snoRNA holds the potential to regulate the activity of the cGAS-STING-IRF3 pathway through this mechanism. As mentioned in the above sections, several snoRNAs interact with cGAS inhibitors such as PARP-1, DNA-PKcs, and CDK1. Would they also participate in innate immunity mediated by the cGAS-STING-IRF3 pathway? Do they regulate the development of lymphocytes in adaptive immune responses? All these questions warrant further exploration.

### snoRNA regulation of cell death signaling pathways

Cells exhibit more than 20 types of cell death patterns, and these are divided into regulatory cell death and accidental cell death. The former is strictly regulated by a series of molecular mechanisms inside the cell (referred to as active cell death) and the latter usually leads to catastrophic cell death due to sudden changes in physical and chemical factors (referred to as passive cell death) [167]. Cell death caused by DNA damage is usually due to a failure of DNA repair that disrupts DNA replication and transcription, chromosome aggregation, or separation processes, causing the passive death of cells (some scholars cautiously propose that this process is not “entirely passive”). Alternatively, the DDR regulatory network triggers programmed death pathways, leading to cell death by several mechanisms, including apoptosis, necroptosis, pyroptosis, autophagy, ferroptosis, and mitotic catastrophe. A programmed death outcome cuts off the iterative expansion of DNA-damaged cells, thereby blocking further damage extension and serving as a self-defense mechanism for cells in which DNA damage has been identified. Liu et al. [168] discussed the involvement of ncRNAs in regulating cell death during tumor metastasis and focused mainly on the roles and mechanisms of several types of ncRNAs, including lncRNAs, miRNAs, and circRNAs in necrotic apoptosis, pyroptosis, and ferroptosis without discussing the influence of snoRNAs on cell death pathways. This section is based on current research knowledge concerning snoRNAs, summarizing the snoRNAs that regulate the process of cell death along with their mechanisms (Table 1 [16, 73, 83, 89, 102, 122, 123, 128, 135, 164, 169–186]). It should be noted that a few lncRNAs (e.g., *LNC-SNO49AB*) containing snoRNA characteristic structures also play a regulatory role in the process of cell death [187]. However, this category of molecules will not be discussed in this section.

**Table 1:** Small nucleolar RNAs (snoRNAs) involved in cell death signaling pathways

**Genes**	**Classification**	**Species**	**Models**	**Primary biological functions**	**Pathways**	**Mechanisms**
*SNORA7B*	H/ACA	Human	Breast cancer [169]	High expression of *SNORA7B* in breast cancer is associated with poor clinical prognosis. Overexpression of *SNORA7B* promotes the proliferation, colony formation, migration, and invasion of breast cancer cells, meanwhile inhibiting cell apoptosis. Conversely, the knockdown of its expression has the opposite effect	Apoptosis	Unclear
*SNORA14A*	H/ACA	Human	Hepatocellular carcinoma (HCC) [135]	*SNORA14A* expression in HCC is downregulated. Overexpression of *SNORA14A* inhibits cell proliferation, and colony formation, induces G2/M phase arrest and cell apoptosis, and inhibits tumor growth in mice	Apoptosis	*SNORA14A* positively regulates the expression of SDHB to regulate succinate metabolism and induces the cleavage of PARP
*SNORA18L5*	H/ACA	Human	HCC [122]	High expression of *SNORA18L5* in HCC is associated with poor prognosis in patients. Overexpression of *SNORA18L5* promotes the proliferation and colony formation of hepatoma cells, promotes G1/S phase transition, inhibits H_2_O_2_-induced apoptosis, and promotes the growth of xenograft tumors in mice	Apoptosis	*SNORA18L5* promotes MDM2-mediated ubiquitination of p53 by regulating the nuclear localization of RPL5 and RPL11, thereby affecting cell cycle progression and apoptosis
*SNORA21*	H/ACA	Human	Gallbladder cancer [128]	*SNORA21* is downregulated in gallbladder cancer and its expression is related to the malignancy of the tumor. Overexpression of *SNORA21* inhibits proliferation, invasion, and migration of gallbladder cancer cells, inducing G1 arrest and apoptosis. Moreover, it suppresses the growth of xenograft tumors in mice	Apoptosis	*SNORA21* induces cleavage activation of caspase 3, increases the expression of BAX, and reduces the expression of BCL2
*SNORA24*	H/ACA	Human	Colorectal cancer [125]	High expression of *SNORA24* in colorectal cancer is negatively correlated with prognosis. Overexpression of *SNORA24* promotes the proliferation and colony formation of colorectal cancer cells, as well as the growth of xenograft tumors in mice; the knockdown of its expression inhibits proliferation and colony formation, and induces cell apoptosis	Apoptosis	The effects of *SNORA24* on apoptosis depend on the p53 signaling pathway. It enhances the degradation of p53 protein via the proteasome pathway, thereby inhibiting the expression of p53
*SNORA38B*	H/ACA	Human	Non-small cell lung cancer (NSCLC) [170]	The high expression of *SNORA38B* in NSCLC is associated with poor prognosis in patients. Overexpression of *SNORA38B* reduces cell apoptosis, and promotes the proliferation, migration, and invasion of lung cancer cells, thus promoting tumorigenesis in mice. *SNORA38B* knockout by CRISPR/Cas9 assay induces an opposite phenotype	Apoptosis, autophagy	It binds with transcription factor E2F1, and negatively regulates the phosphorylation of ULK1 at Ser757 and p53 protein expression through the GAB2/Akt/mTOR pathway, inhibiting autophagy and apoptosis
*SNORA42*	H/ACA	Human	NSCLC [171], prostate cancer [172], HCC [173], colorectal cancer[174]	*SNORA42* is highly expressed in types of cancers, and its expression is negatively correlated with the prognosis of patients. Overexpression of *SNORA42* promotes cell proliferation, invasion, and migration, reduces cell apoptosis, and promotes tumor growth. The knockdown of *SNORA42* has the opposite effect on proliferation, invasion, migration, and cell apoptosis, thus inhibiting tumorigenesis	Apoptosis	*SNORA42* induces the cleavage of caspase 3 and PARP, and its regulation of apoptosis depends on the expression of wild-type p53
*SNORA47*	H/ACA	Human	NSCLC [175], HCC [176]	The high expression of *SNORA47* is associated with the malignant progression of HCC, leading to tumor recurrence and shortening patients’ survival period. The knockdown of *SNORA47* expression inhibits the occurrence of NSCLC and induces cell apoptosis	Apoptosis	*SNORA47* knockdown inhibits the activity of the Akt-ERK pathway, inducing the cleavage of caspase 3
*SNORA71A*	H/ACA	Human	Breast cancer [177]	The highly expressed *SNORA71A* in breast cancer tissues is related to the poor prognosis of patients. Overexpression of *SNORA71A* promotes cell proliferation, invasion, and migration, inhibits cell apoptosis, and promotes xenograft tumor growth in vivo; the knockdown of *SNORA71A* induces the opposite effects	Apoptosis	*SNORA71A* binds with G3BP1, promotes the interaction between G3BP1 and *ROCK2* mRNA, and increases the stability of mRNA molecules to promote the expression of ROCK2 protein
*SNORA71C*	H/ACA	Human	Breast cancer [178]	*SNORA71C* is highly expressed in breast cancer, knockdown of *SNORA71C* inhibits the invasion and metastasis of breast cancer and induces cell death accompanied by an increase of lipid peroxidation product malondialdehyde, and a decrease of antioxidant reductant GSH levels	Ferroptosis	The knockdown of *SNORA71C* inhibits the expression of PTGS2, and GPX4 in breast cancer cells, causing lipid peroxidation to induce ferroptosis
*SNORA74B*	H/ACA	Human	Gallbladder cancer [179]	*SNORA74B* is highly expressed in gallbladder cancer tissues, and its expression is negatively related to clinical prognosis. *SNORA74B* knockdown inhibits the proliferation of gallbladder cancer cells, induces G1 arrest and apoptosis, and suppresses the growth of xenografts in mice	Apoptosis	*SNORA74B* knockdown induces an increase in PHLPP expression which inhibits the activity of the Akt-mTOR pathway, inhibiting the expression of BCL2, but simultaneously enhancing the expression and activity of BAX and caspase 3
*SNORD6*	C/D	Human	Cervical cancer [83]	*SNORD6* is highly expressed in cervical cancer tissues, and its expression is negatively correlated with prognosis. Overexpression of *SNORD6* promotes cervical cancer cell proliferation, invasion, and migration, and inhibits cell apoptosis. The knockdown of *SNORD6* shows the opposite effects	Apoptosis	*SNORD6* binds with the E6 protein and promotes the ubiquitination of p53 through the E6-E6AP-p53 pathway, leading to the degradation of the p53 protein
*SNORD16*	C/D	Human	Colorectal cancer [180]	The high expression of *SNORD16* in colorectal cancer tissues is associated with poor prognosis. Overexpression of *SNORD16* promotes cell proliferation, colony formation, invasion, and migration and inhibits H_2_O_2_-induced cell apoptosis. The knockdown of *SNORD16* induces the opposite effects	Apoptosis	Unknown
*SNORD17*	C/D	Human	HCC [181]	The high expression level of *SNORD17* in HCC is correlated with poor prognosis. *SNORD17* knockdown in p53 wild-type hepatoma cells inhibits cell proliferation and colony formation, causing G1 arrest and cell apoptosis, while overexpression of *SNORD17* promotes cell proliferation and G1/S phase transition, enhancing resistance to doxorubicin-induced apoptosis	Apoptosis	*SNORD17* binds with NPM1, regulating MDM2-mediated ubiquitination of p53 by changing the nucleolar-nuclear distribution of NPM1, negatively regulating the expression of p53 protein
*U32a, U33, U35a*	C/D	Chinese hamster ovary (CHO); mouse	CHO cells, murine myoblasts [16]	The *rpL13a* snoRNAs (*U32a*, *U33*, and *U35a*) are encoded by the *rpL13a* gene. The expression of them is upregulated by lipotoxic drugs such as palmitate and lipopolysaccharides, and they regulate lipid metabolism and H_2_O_2_-induced oxidative stress responses; loss of *rpL13a* alleles or knockdown of *rpL13a* snoRNA reduces palmitate and H_2_O_2_-induced apoptosis	Apoptosis	*rpL13a* snoRNA positively regulates the splicing of *XBP-1* pre-mRNA and the expression of CHOP, inducing cell apoptosis through the ER stress pathway
*SNORD46*	C/D	Human; mouse	Obesity [164]	It is highly expressed in the serum and fat tissues of obese patients/mice, and the *SNORD46* G11A mutation leads to obesity in mice. *SNORD46* inhibits the activity of NK cells in obese patients and increases the toxicity of autophagy inducer on NK cells; *SNORD46* inhibitors antagonize the G11A mutation-induced obesity and enhance the anti-tumor effect of NK cells	Autophagy	*SNORD46* interacts with IL-15, regulating the activity of the IL-15 pathway to inhibit the expression of LC3A/B, thereby suppressing the autophagy of NK cells in obese patients
*SNORD50A/B*	C/D	Human	Breast cancer [123]	The impact of *SNORD50A/B* expression on prognosis in breast cancer patients depends on p53 activity. *SNORD50A/B* deletion in p53 wild-type breast cancer cells inhibits cell proliferation, migration, and invasion, and induces cell apoptosis, but exerts the opposite effects in p53 mutated cells	Apoptosis	*SNORD50A/B* interacts with TRIM21 protein, regulating ubiquitination-mediated degradation of p53 protein through the TRIM21-GMPS pathway. The effect of *SNORD50A/B* on apoptosis depends on the expression of wild-type p53
*SNORD52*	C/D	Human	HCC [89]	The highly expressed *SNORD52* in HCC is associated with poor clinical prognosis. The knockdown of *SNORD52* with ASO induces G2/M arrest and cell apoptosis and inhibits cell proliferation, colony formation, and invasion. It also suppresses tumorigenesis of HCC in mice	Apoptosis	The tumor suppressor gene *Upf1* negatively regulates *SNORD52* expression*. SNORD52* binds with CDK1 to increase its stability and regulates cell cycle and apoptosis depending on CDK1
*U74*, *U81*	C/D	Human	Breast cancer cells, human embryonic kidney cells [182]	Both *U74* and *U81* are encoded by the host gene *Gas5*. Overexpression of *Gas5* inhibits cell proliferation and colony formation but promotes cell apoptosis induced by UV light, dexamethasone, cisplatin, doxorubicin, and okadaic acid	Apoptosis	The induction of apoptosis is regulated by the activity of caspase 8
*SNORD75*	C/D	Human	Breast cancer [78]	*SNORA75* is encoded by the *Gas5* gene, it produces the small ncRNA *pi-sno75*. The expression of *SNORA75/pi-sno75* is downregulated in breast cancer, and *pi-sno75* knockdown increases doxorubicin-induced apoptosis and inhibits the growth of xenograft tumors in mice	Apoptosis	*SNORA75/pi-sno75* promotes the methylation of H3K4 and the demethylation of H3K27 in the promoter region of *TRAIL*, positively regulating TRAIL expression
*SNORD76*	C/D	Human	HCC [183]	*SNORD76* is highly expressed in HCC, and its expression is associated with poor clinical prognosis. Overexpression of *SNORD76* promotes the malignant phenotypes of hepatoma cells including proliferation, invasion, and epithelial-mesenchymal transition. The knockdown of *SNRD76* inhibits cell proliferation and induces G1/S arrest and cell apoptosis, thus inhibiting the growth of xenografts in mice	Apoptosis	*SNORD76* activates the Wnt/β-catenin pathway to regulate cell apoptosis, promoting the onset of HCC
*SNORD78*	C/D	Human	HCC [184], NSCLC [185]	*SNORD78* is encoded by the *Gas5* gene, the expression is upregulated in HCC and NSCLC, and its high expression is associated with the malignant progression of tumors; leading to poor clinical prognosis. Overexpression of *SNORD78* promotes cell proliferation, invasion, migration, epithelial-mesenchymal transition, and self-renewal of cancer stem-like cells. The knockdown of *SNORD78* expression inhibits the malignant phenotype of tumors, induces G0/G1 arrest and apoptosis, and inhibits the growth of xenograft tumors in mice	Apoptosis	The knockdown of *SNORD78* expression reduces the level of BCL2 and increases the expression and activity of BAX, as well as caspase 3
*SNORD88C*	C/D	Human	NSCLC [186]	*SNORD88C* is highly expressed in cancer tissues and patients’ serum, and its level is associated with tumor malignancy. Overexpression of *SNORD88C* promotes malignant phenotype in lung cancer cells, including proliferation, colony formation, invasion, and migration, and reduces the formation of autophagosomes. *SNORD88C* knockdown shows the opposite effect, inducing cell apoptosis and autophagy, and inhibiting the development of tumors in mice	Apoptosis, autophagy	*SNORD88C* regulates the expression and activation of caspase3, positively regulates SCD1 expression by regulating 28S rRNA 2’-O- methylation, and inhibits the transformation of LC3B-I to LC3B-II
*SNORD104* (also known as *U104*)	C/D	Human	Endometrial cancer [102]	*SNORD104* is highly expressed in cancer tissues, and its expression is related to the malignant progression of endometrial cancer. Overexpression of *SNORD104* promotes cell proliferation, colony formation, invasion, and migration, and reduces cell apoptosis, exerting an oncogenic role. Knocking down the expression of *SNORD104* with ASO shows the opposite effects	Apoptosis	*SNORD104* binds with FBL protein to promote the 2’-O-methylation of *PARP-1* mRNA and increases the stability of *PARP-1* mRNA and the expression of PARP-1 protein

*ER* endoplasmic reticulum, *MDM2* murine double minute clone 2, *RPL* ribosomal protein L, *BAX* BCL2 associated X-protein, *BCL2* B-cell lymphoma 2, *GAB* Grb2-associated binder, *AKT* alpha serine/threonine-protein kinase, *mTOR* mechanistic target of rapamycin, *ERK* extracellular signal-regulated kinase, *PARP* poly adenosine diphosphate (ADP)-ribose polymerase, *GPX* glutathione peroxidase, *PTGS2* prostaglandin-endoperoxide synthase 2, *NPM1* nucleophosmin 1, *rpL13a* ribosomal protein L13a, *XBP-1* X-box-binding protein-1, *CDK* cyclin-dependent kinase, *TRAIL* tumour necrosis factor (TNF)-related apoptosis-inducing ligand (TRAIL), *ASO* antisense oligonucleotide, *H*_*2*_*O*_*2*_ Hydrogen peroxide, *GSH* glutathione, *CHOP* C/EBP homologous protein, *SDHB* succinate dehydrogenase subunit B, *CRISPR/Cas9* clustered regularly interspaced short palindromic repeats (CRISPR)/CRISPR-associated nuclease 9, *E2F1* early region 2 factor (E2F) transcription factor 1,* ULK1* unc-51-like autophagy-activating kinase 1, *GAB2* growth factor binding protein 2 (Grb2)-associated binding protein 2,* G3BP1* Ras-GTPase-activating protein SH3 domain-binding protein 1,* ROCK2* rho-associated coiled-coil-containing protein kinase 2,* PHLPP* pleckstrin homology domain leucine-rich repeat protein phosphatases,* TRIM21* tripartite motif-containing protein 21,* GMPS* guanine 5’-monophosphate synthase,* UV* ultraviolet, *H3K4* histone H3 lysine 4,* SCD1* stearoyl coenzyme A desaturase-1,* LC3A/B* light chain 3A/B,* FBL* fibrillarin

#### Apoptosis

Apoptosis is the principal programmed cell death pathway, encompassing three interconnected pathways: intrinsic, extrinsic, and endoplasmic reticulum (ER) stress pathways. (1) The intrinsic pathway (also known as the mitochondrial pathway) is triggered by various microenvironmental disturbances, including DNA damage, oxidative stress, and replication stress. The central process in this pathway involves permeabilization of the mitochondrial outer membrane, resulting in the release of cytochrome C and subsequent cleavage and activation of the caspase protein family. This process is dynamically regulated by pro-apoptotic proteins from the B-cell lymphoma 2 (BCL2) family [BCL2 associated X-protein (BAX), BCL2 antagonist/killer 1 (BAK), BCL2 ovarian killer (BOK), BH3-interacting domain death agonist (BID), p53 upregulated modulator of apoptosis (PUMA), BCL2 interacting mediator of cell death (BIM), and NADPH oxidase activator (NOXA)], as well as anti-apoptotic proteins [BCL2, B-cell lymphoma-extra large (BCL-XL), myeloid leukemia cell differentiation protein-1 (MCL-1), BCL2-like protein 2 (BCL2L2), and BCL2-related protein A1 (BCL2A1)]. (2) The extrinsic pathway (also known as the death receptor pathway) is mediated by “death receptors” such as fatty acid synthase (FAS), tumor necrosis factor (TNF) receptor 1 (TNFR1), tumor necrosis factor receptor 2 (TNFR2), TNF-related apoptosis-inducing ligand receptor 1 (TRAILR1), and TNF-related apoptosis-inducing ligand receptor 2 (TRAILR2) which are activated by extracellular ligands. The activation of these death receptors leads to caspase 8 and caspase 10 activation followed by apoptosis. (3) In the ER-induced apoptosis, various injurious factors lead to an imbalance of Ca^2+^ ions in the ER cavity, resulting in increased misfolded or unfolded proteins [188]. p53 is involved in DNA damage-induced apoptosis. Proteins like ATM, ATR, and DNA-PKcs phosphorylate p53 at serine 15 and serine 20, thereby enhancing its transcriptional activity on downstream genes and ultimately promoting the expression of pro-apoptotic proteins such as NOXA (encoded by *PMAIP1*) and PUMA (encoded by *BBC3*) [189]. CDKs also play a crucial regulatory role in the expression and modification of BCL2 family proteins. The expression of the anti-apoptotic factor MCL-1 is significantly inhibited in apoptotic cells treated with the pan-CDKs inhibitor flavopiridol, while the expression levels of BIM, NOXA, and BCL2-interacting killer (BIK) are increased [190, 191].

The regulation of cell apoptosis is a critical focus in snoRNA research in the field of cell biology. To our knowledge, the p53 signaling pathway serves as the primary mechanism through which snoRNAs regulate apoptosis. For example, *SNORA42*, *SNORD50A/B*, *SNORA24*, *SNORD6*, *SNORD17*, *SNORA18L5*, and *SNORA38B* exert their effects on apoptosis that strictly depend on the expression and activity of the p53 protein [83, 122, 123, 125, 170, 171, 181]. Additionally, snoRNAs also target members of the BCL2 family to regulate apoptosis. By inhibiting BCL2 and simultaneously increasing BAX protein expression levels, both *SNORD78* and *SNORA74B* promote apoptosis [179, 185]. Conversely, *SNORD75* promotes the transcription of the pro-apoptotic gene *TRAIL* by regulating the methylation levels within its promoter region [78]. It should be noted that the DNA repair molecule PARP-1 and the cell cycle checkpoint protein CDK1 participate in the snoRNA-mediated regulation of apoptosis [89, 102]. Furthermore, bioinformatics analysis has provided additional insights into snoRNA-mediated regulation of apoptosis. By analyzing data from 130 patients with AML derived from The Cancer Genome Atlas (TCGA) website, researchers identified 14 snoRNA molecules that effectively predict AML risk (high or low). Gene Set Enrichment Analysis (GSEA) revealed significant enrichment of these 14 snoRNAs in both cell apoptotic pathways and AML-related signaling pathways [192]. Taken together, this evidence suggests that the regulation of apoptosis represents an important pathway through which snoRNAs exert their function. However, it is still unclear whether snoRNAs related to apoptosis are involved in the DDR-induced cell apoptotic process.

#### Autophagy

Autophagy is a stress-adaptive mechanism that occurs in cells experiencing various stress conditions, including starvation, hypoxia, and DNA damage. The autophagy process involves the formation of autophagosomes and autolysosomes with the specific purpose of degrading cellular proteins and organelles, thereby enabling the conservation of energy metabolism and cellular renewal. Autophagy has traditionally been viewed as a protective mechanism promoting cell survival by reducing the toxicity of certain DNA-damaging drugs in tumor cells [193, 194]. However, it has been recently discovered that autophagy is associated with cell death, a process referred to as “autophagy-dependent cell death”. Although the molecular mechanism underlying this form of cell death is not fully elucidated, it shares common signaling pathways with canonical autophagy. Key regulators driving this process include conserved ubiquitin-fold proteins [e.g., autophagy-related (ATG) proteins]. Among them, the ULK1/2 complex (homologous to yeast ATG1) plays a pivotal role in promoting autophagosome formation. Additionally, ATG4 and ATG7 are critical for cleaving and activating light chain 3 (LC3), leading to its transformation from LC3-I to LC3-II (a hallmark of autophagy) [193, 195]. The activation of proteins such as ATM, ATR, c-Jun N-terminal kinase (JNK), and PARP-1 during DDR, along with subsequent activation of their substrates [adenosine monophosphate (AMP)-activated protein kinase (AMPK), mTOR, p53, and BCL2] regulate the expression and modification of ULK and Beclin 1 (homologous to yeast ATG6), ultimately promoting autophagy-dependent cell death [196, 197]. It is also worth noting that cellular autophagy induced by stress-adaptive responses reciprocally influences the DNA damage repair process [198].

Since 2022, Zhuo et al. [170], Wang et al. [186], and Zhang et al. [164] have independently demonstrated the direct involvement of *SNORA38B*, *SNORD88C*, and *SNORD46* in the regulation of autophagy. Zhuo et al. [170] found that *SNORA38B* binds to the transcription factor early region 2 factor (E2F) transcription factor 1 (E2F1) and promotes ULK1 phosphorylation at the Ser757 site through the growth factor binding protein 2 (Grb2)-associated binding protein 2 (GAB2)/Akt/mTOR pathway. The phosphorylation of the ULK1 at the Ser757 site inhibits the interaction between ULK1 and AMPK, subsequently leading to the suppression of autophagy-induced apoptosis [197]. Wang et al. [186] investigated the impact of *SNORD88C* on autophagosome formation using fluorescence microscopy and transmission electron microscopy. The authors observed that the knockdown of *SNORD88C* expression significantly increases the number of autophagosomes, whereas *SNORD88C* overexpression reduces autophagosome formation. Moreover, molecular evidence supporting cell-autonomous inhibition of autophagy by *SNORD88C* was provided through analysis of protein expression levels for LC3-I, LC3-II, and p-ULK1 Ser757. In 2023, Zhang et al. [164] reported that *SNORD46* inhibits autophagy in NK cells by regulating the activity of the IL-15 pathway and suppressing light chain 3A and 3B (LC3A/B) expression levels. Moreover, a G11A mutation in *SNORD46* was shown to increase the toxic reaction of NK cells towards tuberostemonine as an inducer for autophagy. To date, no other snoRNAs have been reported as the regulator involved in either autophagy or processes related to cell death dependent on this process.

#### Ferroptosis

Ferroptosis, initially discovered by Dixon et al. [199] in 2012, is a form of iron-dependent regulatory cell death, characterized by reduced or vanished mitochondrial cristae, rupture of the outer mitochondrial membrane, mitochondrial shrinkage, and increased membrane density. It primarily involves abnormal metabolism of lipid peroxides catalyzed by iron ions, ultimately leading to cell death due to disruption of redox homeostasis. Despite its crucial role in various clinical diseases, the underlying molecular mechanisms of ferroptosis remain unclear. Current knowledge suggests that factors such as the expression and activity of the cystine/glutamate transporter system x_c_^-^ (SXC), reduced GSH levels, the activity of GSH-dependent enzyme glutathione peroxidase 4 (GPX4), and cellular ability to acquire iron ions (Fe^2+^) all regulate ferroptosis [200]. Iron ions in cells generate hydroxyl free radicals (OH•) through the Fenton reaction [Fe^2+^ + H_2_O_2_ → Fe^3+^ + (OH)^-^ + OH•], which depletes hydrogen atoms between the long-chain double bonds in polyunsaturated fatty acids on the biofilm and induce lipid peroxidation [201]. GPX4 serves as a “molecular switch” utilizing GSH to reduce lipid peroxides and protect cells from ferroptosis. The depletion of GSH or inactivation of GPX4 caused by exogenous drugs increases the generation of lipid peroxides, leading to ferroptosis [167].

To date, only one article has reported on the involvement of snoRNAs in the process of ferroptosis. In 2023, Xie et al. [178] demonstrated that the downregulation of *SNORA71C* expression in breast cancer cells exerted an inhibitory effect on the expression of GPX4 and prostaglandin-endoperoxide synthase 2, resulting in increased levels of the lipid peroxidation product malondialdehyde and reduced GSH levels, eventually leading to ferroptosis. As SXC regulates the interexchange of glutamate and cystine inside cells, its ability to acquire cystine directly influences its antioxidant capacity. Jiang et al. [202] discovered that p53 inhibits the expression of *SLC7A11*, a component of SXC, thereby enhancing cell sensitivity to ferroptosis inducers (such as elastin). However, it remains unknown whether snoRNA genes involved in the p53 signaling pathway have an impact on ferroptosis or how snoRNA-mediated regulation contributes to DDR-induced ferroptosis.

## Significance of snoRNA in disease diagnosis and treatment

The role of snoRNA deletion, mutation, and genetic imprinting in the pathological processes of human diseases has been a subject of interest for many years. PWS is the earliest disease to be recognized as a rare genetic disorder caused by defects in genomic imprinting in the 15ql1q13 region, involving the deletion of *MBII*-*52* (also known as *SNORD115*) and *MBII-85* (also known as *SNORD116*) [13]. Leukoencephalopathy with brain calcifications and cysts (LCC, also known as Labrune syndrome) is the first disease attributed to autosomal recessive mutations in a snoRNA gene (*SNORD118*, also known as *U8*) [17, 203]. The advancement in the research on snoRNA function has revealed that an increasing number of snoRNAs are dysregulated in clinical diseases, with their biological functions substantially interrelated with the pathological processes of these diseases. In this article, we utilized the MNDR v2.0 website (www.rna-society.org/mndr/) established by Cui et al. [204] to systematically analyze snoRNA-related diseases, and more than 60 have been confirmed as snoRNA-related diseases to date. The main categories of these diseases include cancer, immune diseases, genetic developmental disorders, infections, abnormal hyperplasia, cardiovascular conditions, metabolic disorders, and others, with cancer accounting for over half of them. These diseases involve hundreds of snoRNAs forming a gene pool with promising clinical prospects (with some possibility that a few molecules may have been overlooked). In recently published reviews, several researchers discussed the relationship between snoRNAs and clinical diseases associated with genomic instability [3, 205–207]. These diseases encompassed a range of malignant tumors (HCC, colorectal carcinoma, breast cancer, leukemia, lung cancer, prostate cancer, and gliomas); genetic disorders (PWS, LCC, myelodysplastic syndromes, and X-linked dyskeratosis congenital); as well as neurodegenerative conditions (Alzheimer’s disease and Huntington’s disease). Additionally mentioned were psychiatric disorders (autism spectrum disorder); cardiovascular diseases (congenital heart disease, cardiometabolic disease, hypertrophic cardiomyopathy, heart failure, myocardial infarction, peripheral artery disease, and hypertrophic cardiomyopathy); along with inflammatory, immune, and metabolic disorders [3, 17, 159, 203, 205–215]. Interestingly, some of the snoRNA molecules discussed here have been identified as potential diagnostic biomarkers or therapeutic targets that hold promise for clinical applications.

## Conclusions and perspectives

Significant progress has been made in the past decade regarding the exploration of the biological functions associated with snoRNAs. The primary focus has centered on investigating the pro- or anti-oncogenic roles of snoRNAs in various processes, including tumorigenesis, invasion, metastasis, and drug resistance. Despite these notable strides, current research has primarily concentrated on elucidating the fundamental functions of snoRNAs within specific biological processes, leaving the underlying molecular mechanisms largely unclear. This review provides a comprehensive summary of the functional mechanisms attributed to snoRNAs and suggests that although there is evolutionary conservation in their interactions with RNAs/proteins, some diversity exists between species and tissues. Furthermore, compared to their classical role in regulating modifications of rRNAs and snRNAs, much remains unknown about how snoRNAs regulate mRNA and tRNA modifications. It is also important to note that interactions between snoRNAs and protein molecules are likely to significantly streamline the regulatory pathway through which snoRNAs exert their function.

**Fig. 8:**
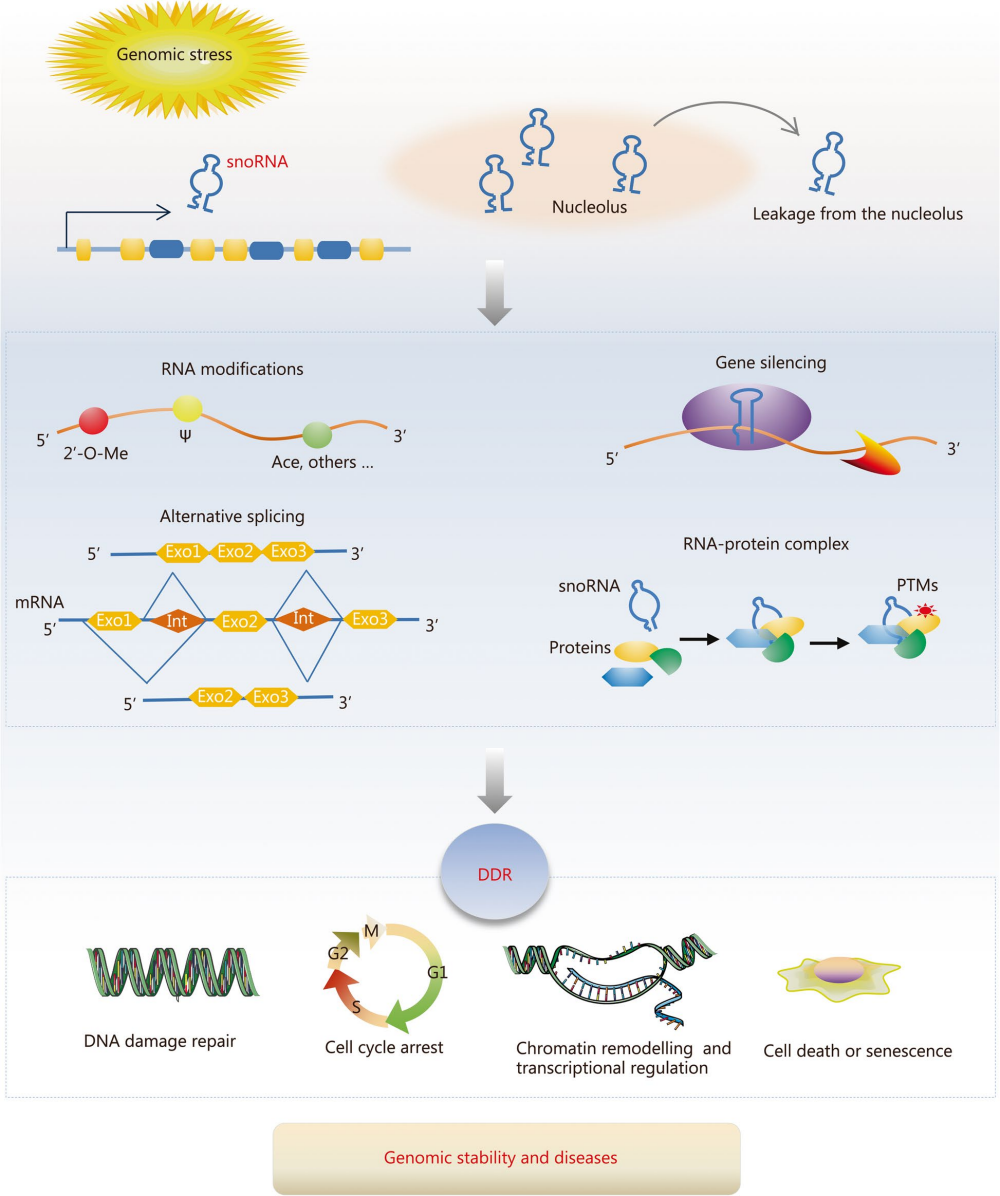
A framework of snoRNA in mediating genomic stress-induced DDR. snoRNA small nucleolar RNAs, 2’-O-Me 2’-O-methylation, ψ pseudouridylation, Ace acetylation, mRNA messenger RNA, Exo exon, Int intron, PTM post-translational modification, DDR DNA damage response, G1 gap1 phase, S synthesis phase, G2 gap2 phase, M mitotic phase

Understanding the regulatory mechanisms of DDR is vital for human health, as it facilitates the identification of underlying causes of critical diseases and guides the development of treatment approaches. To date, various inhibitors targeting DDR pathways have been applied in patients, including the well-known PARP-1 inhibitor that effectively induces synergistic lethality in tumor cells with HR repair defects by inhibiting SSB repair [2]. This review primarily focuses on elucidating the regulatory roles of snoRNAs in DDR under diverse genomic stresses. Current research findings about snoRNA-mediated regulation of oxidative stress, DNA damage repair, cell cycle checkpoint activation, cell death, immunity, and inflammation, are comprehensively collected and summarized to establish a theoretical framework for understanding “the role of snoRNA in mediating DNA damage repair” (Fig. 8).

As our understanding of the functions of snoRNAs deepens, their roles in the regulatory network of DDR will gradually be established. The findings summarized in this review are expected to pave the way for a new research area in the future. Although several snoRNAs (including *SNORA73A/B*, *scaRNA2*, *SNORD50A/B*, and *SNORA24*) have been shown to regulate the expression and activity of key molecules involved in DNA damage repairs such as PARP-1, DNA-PKcs, and p53, it is important to note that these few snoRNAs do not provide a comprehensive representation of the entire snoRNA family [18, 22, 123]. Therefore, addressing the following crucial questions is necessary to gain a complete understanding of this research field.

(1) Do snoRNAs synergistically regulate DDR through multiple signaling pathways? The mechanisms regulating snoRNA function are complex, as they typically enable a wide range of modifications to various RNA molecules, including rRNAs, snRNAs, mRNAs, and tRNAs. Simultaneously, they generate numerous regulatory sdRNAs with biological activity. Moreover, snoRNAs regulate gene expression at multiple levels encompassing transcription, post-transcription, translation, and post-translation modification. A study on the role of *scaRNA2* in DNA damage repair suggests that it interferes with the interaction between DNA-PKcs and KU protein (inhibiting the formation of the DNA-PK complex), while also competitively binding to the lncRNA *LINP1* (a gene-enhancing DNA-PKcs activity). Indeed, these two pathways work synergistically to inhibit the kinase activity of the DNA-PK complex [22]. Thus, it can be inferred that the regulation of the DNA damage signal network by snoRNAs may not rely solely on a single pathway; instead, the application of multiomics technology may be necessary to fully elucidate its underlying molecular mechanism.

(2) Do snoRNAs function as mediators connecting ribosome biogenesis stress and nucleolar stress response to DDR? Ribosome biogenesis primarily occurs in the nucleolus and is highly sensitive to various internal and external stimuli. Disturbances in transcription, processing, and modification of rRNA, or assembly of ribosomal subunits induce a nucleolar stress response, leading to cellular stress adaptive responses or the activation of cellular death pathways. Additionally, the nucleolus is an important stress sensor in the cell. DNA damage caused by UV radiation, ionizing radiation, and chemotherapeutic drugs (such as platinum-based drugs) inhibit the transcriptional activity of RNA polymerase on rDNA in an ATM-dependent manner, resulting in nucleolar reorganization, recruitment of various DNA damage repair proteins to the nucleolus (including ATM, DNA-PKcs, and PARP-1), and activation of the p53 pathway [216–220]. Although the interaction between nucleolar stress and DDR is well recognized, it was previously believed that disturbances in ribosome biogenesis and nucleolar stress response were events downstream of DNA damage. However, recent research has revealed a more intricate reality than previously understood. Multiple enzymes involved in ribosome biogenesis [including apurinic/apyrimidinic endonuclease 1 (APEX1), WRN, and BLM] are now known to participate in various DNA repair pathways such as BER, HR, and NHEJ [221]. Moreover, pre-rRNA plays a role in recognizing DNA damage sites during meiosis and functions as a molecular scaffold to facilitate the recruitment of different repair molecules [222]. Although snoRNAs are known regulators of nucleolar function, the mechanism by which they modulate ribosome biogenesis in DDR remains enigmatic. Limited studies have suggested that the interaction between snoRNAs and DNA repair molecules promotes ribosome biogenesis. In a KU protein-dependent manner, DNA-PKcs regulates 18S rRNA processing, thereby promoting bone marrow hematopoiesis through the regulation of ribosome biogenesis. The core mechanism underlying this biological function is the *U3*-mediated regulation of the phosphorylation at Thr2609 on DNA-PKcs [15]. Additionally, *SNORA73* activates PARP-1 by regulating its autoPARylation and facilitates the aggregation of DDX21 in the nucleolus to drive rDNA transcription and ribosome biogenesis [100]. Exploring whether snoRNAs serve as mediators to synchronize the processes involved in ribosome biogenesis, nucleolar stress, and DNA damage repair is an important question worth investigating. The connection established by snoRNAs may provide crucial evidence for understanding both the early events in DNA damage-induced nucleolar stress and the outcomes observed in damaged cells.

(3) Do snoRNAs participate in the cellular response to radiation damage by regulating DDR? The development and application of nuclear technology have increased the population at risk of ionizing radiation, particularly in clinical medical procedures. Radiation therapy is widely applied in the treatment of diseases, with over 50% of patients with cancer receiving it as part of their treatment plans. However, ionizing radiation induces nuclear DNA damage in cells. The sensitivity of organisms and cells to DNA damage, as well as their repair capacity, directly influences the severity of radiation-induced damage and the risk of carcinogenesis. Importantly, individual radio-sensitivity significantly impacts patient prognosis due to variations in radiotherapy tolerance or severe adverse reactions [223]. As mentioned in this article, the regulatory role of snoRNAs in DDR has recently gained considerable attention from researchers. However, there is currently limited evidence supporting the involvement of snoRNAs in regulating the response to radiation-induced damage. Nonetheless, a few research teams have made preliminary explorations in this field. For example, Huo et al. [224] observed that the exposure of BmN4 cells to UV radiation leads to the nuclear-cytoplasmic shuttling of the C/D box snoRNA *Bm-15* and its subsequent accumulation in the cytoplasm. Additionally, Rastorgueva et al. [90] analyzed snoRNA expression profiles at different time points after the X-ray irradiation in radiation-sensitive and radiation-tolerant leukemia cell lines, leading to the identification of several genes potentially associated with radiation damage. However, the authors did not perform a functional validation of the specific molecules involved. So far, *scaRNA2* has been confirmed as a snoRNA molecule exerting significant regulatory effects on radiation-induced DDR [22, 87]. Given that the pool of snoRNA represents a potential molecular reservoir for regulating DDR, exploring their functions and mechanisms during the radiation damage process may provide novel biomarkers and therapeutic targets for assessing and intervening in radio-sensitivity.

In conclusion, investigating the functions and mechanisms of snoRNAs will greatly enrich the regulatory network of ncRNAs. snoRNAs can be regarded as crucial participants in the process of DNA damage repair, thus forming a promising new field of research. Finally, snoRNAs possess unpredictable potential applications in fields such as radiation damage and tumor radiation/chemotherapy.

## Data Availability

Not applicable.

## Abbreviations

5-HT_2C_R: 5-hydroxytryptamine 2C receptor
ADP: Adenosine diphosphate
A-I editing: Adenine to inosine editing
AML: Acute myeloid leukemia
ATG: Autophagy-related
ATM: Ataxia telangiectasia mutated
ATR: ATM and Rad3-related
BER: Base excision repair
CDC25: Cell division cyclin 25
CDK: Cyclin-dependent kinase
cGAS: Cyclic guanosine monophosphate-adenosine monophosphate synthase
CKI: CDK inhibitor
CSR: Class switch recombination
DDR: DNA damage response
DNA-PK: DNA-dependent protein kinase
DNA-PKcs: DNA-dependent protein kinase catalytic subunit
DSB: Double-strand break
dsDNA: Double-stranded DNA
ER: Endoplasmic reticulum
FAT: FRAP-ATM-TRRAP
GPX4: Glutathione peroxidase 4
GSH: Glutathione
H_2_O_2_: Hydrogen peroxide
HCC: Hepatocellular carcinoma
HR: Homologous recombination
IFN: Interferon
IRF3: IFN regulatory factor 3
LncRNA: Long non-coding RNA
MDM2: Mouse double minute 2
miRNA: MicroRNA
mRNA: Messenger RNA
MRN: MRE11-RAD50-NBS1
ncRNA: Non-coding RNA
NHEJ: Non-homologous end joining
nt: Nucleotides
2’-O-Me: 2’-O-methylation
PARP-1: Poly (ADP-ribose) polymerases-1
PARylation: Poly-ADP-ribosylation
PIKKs: Phosphatidylinositol 3-kinase-related kinase family members
piRNA: Piwi-interacting RNA
PTM: Post-translational modification
PWS: Prader-Willi syndrome
rDNA: Ribosomal DNA
RNS: Reactive nitrogen species
ROS: Reactive oxygen species
rpL13a: Ribosomal protein L13a
rRNA: Ribosomal RNA
scaRNA: Small Cajal body-specific RNA
sdRNA: Sno-derived RNA
snoRNA: Small nucleolar RNA
snoRNP: Small nucleolar ribonucleoprotein
snRNA: Small nuclear RNA
snRNP: Nuclear ribonucleoprotein
SSB: Single-strand break
ssDNA: Single-stranded DNA
STING: Stimulator of interferon genes
SXC: System x_c_
tRNA: Transfer RNA
UV: Ultraviolet
V(D)J: Variable (diversity) joining
